# A review of travel and arrival-time prediction methods on road networks: classification, challenges and opportunities

**DOI:** 10.7717/peerj-cs.689

**Published:** 2021-09-08

**Authors:** Asad Abdi, Chintan Amrit

**Affiliations:** 1 Department of Industrial Engineering and Business Information Systems, Behavioural, Management & Social Sciences, University of Twente, University of Twente, Enschede, Netherlands; 2 Department of Operations Management, Amsterdam Business School, University of Amsterdam, Amsterdam, Netherlands

**Keywords:** Intelligent transportation systems, Travel time prediction, Arrival time prediction, Spatial-temporal features

## Abstract

Transportation plays a key role in today’s economy. Hence, intelligent transportation systems have attracted a great deal of attention among research communities. There are a few review papers in this area. Most of them focus only on travel time prediction. Furthermore, these papers do not include recent research. To address these shortcomings, this study aims to examine the research on the arrival and travel time prediction on road-based on recently published articles. More specifically, this paper aims to (i) offer an extensive literature review of the field, provide a complete taxonomy of the existing methods, identify key challenges and limitations associated with the techniques; (ii) present various evaluation metrics, influence factors, exploited dataset as well as describe essential concepts based on a detailed analysis of the recent literature sources; (iii) provide significant information to researchers and transportation applications developer. As a result of a rigorous selection process and a comprehensive analysis, the findings provide a holistic picture of open issues and several important observations that can be considered as feasible opportunities for future research directions.

## Introduction

Transportation provides several benefits to the world in terms of worldwide cargo delivery, providing access to people, enhancing the quality of life and the overall well-being of the economy and environment. On the other hand, it presents significant challenges including fuel consumption, traffic congestion, carbon emissions, economic and environmental costs. Hence, there is a need to create an effective and sustainable transportation system that can assist and allow companies, planners, policymakers, etc, to determine the best solution to transportation problems. The advanced developments of information technology (IT) have provided remarkable occasions to enhance the performance of transportation system (TS). On the other hand, the availability of a large transportation dataset and the ability to process a significant amount of data has attracted lots of attention in recent years to research in the area of transportation modelling for various purposes (**e.g*., arrival time prediction (ATP), travel time prediction (TTP)*) (Du, Peeta & Kim, 2012).

However, recent technological advancements have caused a widespread uptake of various technologies in the field of transportation, *e.g.*, Intelligent Transportation Systems (ITS), which are intended to tackle the aforementioned problems. It is worth noting here that an ITS is also useful for passengers (*because of the anxieties, satisfaction, waiting times*) and transport services (*because of the user-friendly, competitiveness among various transportation modes, reliability, etc*.). The remarkable advancements of technologies in the last decades have led to more and more ITS being developed and implemented. On the other hand, despite the advancement of information technology, there are still several outstanding issues in this area. Thus, considering this wide variety of deployment, there is a need to highlight the remaining drawbacks, outline and analyze the current state of the field (Choudhary, Khamparia & Gahier, 2016).

This study provides a new systematic literature review (SLR) to have a holistic picture of ATP and TTP models on the road network. For this reason, the current work aims to ***(i)*** prepare a structured and broad overview of the existing methods to indicate transportation deficiencies and drawbacks mainly in travel time and arrival time modelling on road transportation; ***(ii)*** Classify the existing methods, describe the essential concepts, provide a description of various factors that influence the travel time/arrival time, provide an explanation of different dataset resources and the characteristics of the datasets, create a comprehensive background analysis of the existing method and an analysis of various evaluation metrics involved in travel time and arrival time modelling; ***(iii)*** identify challenges associated with the techniques, and to ***(iv)*** discuss some of the future works to improve the performances of existing methods.

There have been some studies that aimed to review the literature that tackles the topic of ATP and TTP. For instance, Choudhary, Khamparia & Gahier (2016) aimed to provide a detailed description of various studies conducted on bus arrival time from 2004 to 2015. This study presents various techniques such as SVM, fuzzy logic, KNN, Kalman filter, hybrid models, etc. In another review paper by Nguyen et al. (2018) reviewed only the deep learning-based approaches for popular issues in the transportation domain such as “*traffic flow forecasting*”, “*autonomous driving*”, etc. Nguyen et al. (2018) present an overview of deep learning structures (*e.g*., RNN (Graves, Mohamed & Hinton, 2013), CNN (Simonyan & Zisserman, 2014), RBM (Goodfellow et al., 2013), AE (Gehring et al., 2013), DBN (Sarikaya, Hinton & Deoras, 2014) and LSTM (Sainath et al., 2015)) as well as their applications in popular traffic data analytics.

Mori et al. (2015) aimed to present an overview of the existing methods to estimate travel time (their estimation method computes travel times of trajectories using data captured during the journey (Bovy & Thijs, 2000) and travel time prediction (*a prediction method to forecast travel time using the current and the past data*), which are two primary problems in travel time modelling. The paper also explains the dataset that can be employed in various approaches such as point detectors and interval detectors. Furthermore, it indicates the limited number of factors that affect the punctuality of ATP and estimation approaches. Similarly, Bai et al. (2018) conducted a review focusing on travel-time prediction methods and provided a brief explanation of some methods such as linear regression (LR), Kalman filter (KF), SVR, etc. They compared these methods in terms of their accuracy for a different dataset. Furthermore, Abduljabbar et al. (2019) presented different applications of Artificial intelligence (AI) approaches applied to various transport-related challenges. Their study aimed at enhancing the planning, decision making and management of road traffic (**e.g*., signal traffic control, incident detection, traffic management, intelligent urban mobility, etc*.). Altinkaya & Zontul (2013) also presented a survey on various methods based on historical data, KF, statistical methods and ANN that have been proposed for ATP.

This study makes a significant contribution compared to the aforementioned papers due to the following reasons:

***(i)*** Our work is one of the first efforts to focus on both TTP and ATP, while other works (*e.g*., (Choudhary, Khamparia & Gahier, 2016; Altinkaya & Zontul, 2013) (excluding travel time), (Mori et al., 2015; Bai et al., 2018) (excluding arrival time)) do not consider both approaches. Furthermore, in this study, we did not focus only on one aspect of the relevant approaches (**i.e*., deep learning*), but we discuss a taxonomy of various methods. Moreover, the current work illustrates comparative information of the techniques, evaluation metrics, datasets resources, etc. in the form of graphs and tables.

***(ii)*** Our survey includes papers from 2010 to 2021, while other existing review papers (*e.g*., Choudhary, Khamparia & Gahier, 2016) includes papers published between 2004 and 2015, (Altinkaya & Zontul, 2013) consists of papers published until 2013, (Nguyen et al., 2018), (Bai et al. 2018), (Abduljabbar et al., 2019) contains papers published until 2018; (Mori et al. 2015) contains papers published until 2014 and do not include recently published papers.

***(iii)*** Our review paper indicates several factors that affect the punctuality of ATP and TTP models, while other existing papers (*e.g*., Choudhary, Khamparia & Gahier, 2016; Nguyen et al., 2018; Abduljabbar et al., 2019; Abduljabbar et al., 2019; Altinkaya & Zontul, 2013) do not indicate these factors. Especially the papers presented by Mori et al. (2015) and Bai et al. (2018) only contain a limited number of factors.

***(iv)*** Our work explains several datasets resources that can be utilized to illustrate the various methods, while other existing papers (*e.g*., Choudhary, Khamparia & Gahier, 2016; Nguyen et al., 2018; Abduljabbar et al., 2019; Altinkaya & Zontul, 2013; Bai et al., 2018) do not present any data sources. Mori et al. (2015) describe two types of data sources such as point detectors and interval detectors.

***(v)*** Our study lists several performance measures, while other papers (*e.g*., Choudhary, Khamparia & Gahier, 2016; Nguyen et al., 2018; Abduljabbar et al., 2019; Altinkaya & Zontul, 2013) do not mention them. Bai et al. (2018) describe a few performance measures.

***(vi)*** Finally, unlike other surveys, our work explains the strengths and limitations of the proposed methods.

However, this study not only complements earlier review papers but is provide an in-depth insight into ATP and TTP models. The present study takes account of different aspects such as the type of dataset, evaluation metrics, factors, proposed methods and their advantages and disadvantages, etc. Moreover, bringing useful information and knowledge on different aspects of ATP and TTP models could be beneficial for system developers, and might be a source of inspiration in using new methods and approaches typical for a certain purpose. The finding of this study will be relevant for researcher and developer in the transportation domain. It also indicates the promising paths for future research.

The rest of this paper is structured as follows: ‘Survey Methodology’ presents the research questions, the selection criteria, and the search process. ‘Analysis and Discussion’ is subdivided into the travel and arrival time modelling, The influence factors, dataset resources and evaluation metrics. Finally, the paper discusses the main findings and limitations of the study, as well as provides general recommendations.

## Survey Methodology

### Research method

The methodology employed in this study is the systematic literature review (SLR). A literature review contains a set of processes to identify as well as interpret all the works to provide answers to pre-defined research questions, gaps in the existing researches and draw conclusions based on the research questions (Kitchenham, 2004). The PRISMA (http://www.prisma-statement.org/) (*Preferred Reporting Items for Systematic Reviews and Meta-Analyses*) was employed as the guidelines to conduct and report this review work. In the following sub-sections, we discuss in detail the designed review strategy, which includes the research questions, the selection criteria and the selection process, search strategy and presentation.

### Research questions

We formulated the following research questions (RQ) to achieve the aim of this study.

1. What techniques are being used to model travel time and arrival time prediction on road?
2. What are the challenges associated with the existing techniques?
3. How can the various evaluation metrics, dataset resources and factors involved in transportation problems (*travel and arrival time modelling*) be analysed?
4. How could feasible opportunities be highlighted for future research work?

The first and second research questions are addressed in sections ‘*A taxonomy of the ATP and TTP methods*’ and ‘*Strengths and Weaknesses*’, in which we have identified numerous techniques and provide a broad overview of them. We also classify these techniques and discuss the challenges associated with them. It is important to note that Research Question one implies that this review is focused exclusively on the ATP and TTP models on road networks. Concerning research question (three), sections ‘*The factors contributing to ATP and TTP*’, ‘*Dataset collection systems*’ and ‘*Evaluating ATP and TTP approaches*’ provides a broad overview of factors that are used to model arrival and travel time prediction models as well as the evaluation metrics used in this field. ‘Future work’, research question (four), presents several issues as opportunities for future research work.

### Search process

To obtain eligible studies various databases (*e.g*., IEEE-explore, Google Scholar, *ACM Digital Library, Science Direct, Springer, SAGE, Web of Science (formerly known as ISI Web of Knowledge), etc.)* were searched online for journal papers and conference proceedings (the search timeline is between 2010 and 2021). For this reason, we collected several research terms and their corresponding synonymous words (*e.g*., “*arrival time*”, “*travel time*”, “*journey time*”, “*method*”, “*approach*”, “*algorithm*”, etc.). Next, to construct a search query that can yield the most relevant and complete list of papers, we performed several search iterations. We searched on the corresponding databases using search strings to obtain relevant papers. To create a search string, we also used the Boolean “*OR*” and “*AND*” operators. The Boolean OR operator is used to include substitutes and alternative words, while the Boolean AND is used to connect and define the relationship between the search terms. For instance, the following query was used as a search string.

((“*travel time*” OR “*journey time*”) OR (“*arrival time*”) AND (“*prediction*” OR “*estimation*” OR “*forecasting*”) AND (“*approach*” OR “*technique*” OR “*method*” OR “*algorithm*”)).

### Inclusion and exclusion criteria

In this phase of our SLR, the retrieved papers were reviewed by reading the title, abstract and conclusion. We also considered the inclusion and exclusion criteria to discard irrelevant papers based on their titles and abstracts. However, we list some inclusion and exclusion criteria on which papers can be included as well as those that need to be excluded. We presented a list of eligible publications based on papers that satisfied at least one of the inclusion criteria. Likewise, a paper was excluded if it matched any of the following exclusion criteria.

Inclusion Criteria: papers were included based on the following criteria.

- Peer-reviewed papers published from 2010 to 2021.
- The paper must include a technique to model either ATP or TTP.
- It needs to answer at least one of the research questions.
- Available in full text.

Exclusion Criteria: papers were excluded based on the following criteria.

- Abstracts, editorials and unpublished material.
- Non-English or publications having no English translation are also not considered.
- Duplicate reports of the same paper (*the most complete version or the most recent report is selected*).
- The study does not provide sufficient details to understand the system architecture and design.

### Quality assessment criteria

In addition to the aforementioned criteria, the quality assessment criteria were also applied to each paper to select papers that would be able to provide reliable and high-quality answers to the research questions. However, we consider the following criteria to assess the reliability and quality of the selected papers:

- Are the aims of the research presented clearly?
- Is the proposed method clearly explained?
- Is the experimental design appropriate?
- Is the method validated through an adequate number of project data sets or case studies?
- Does the research add value to the academic community?

### Selection

**Figure 1:**
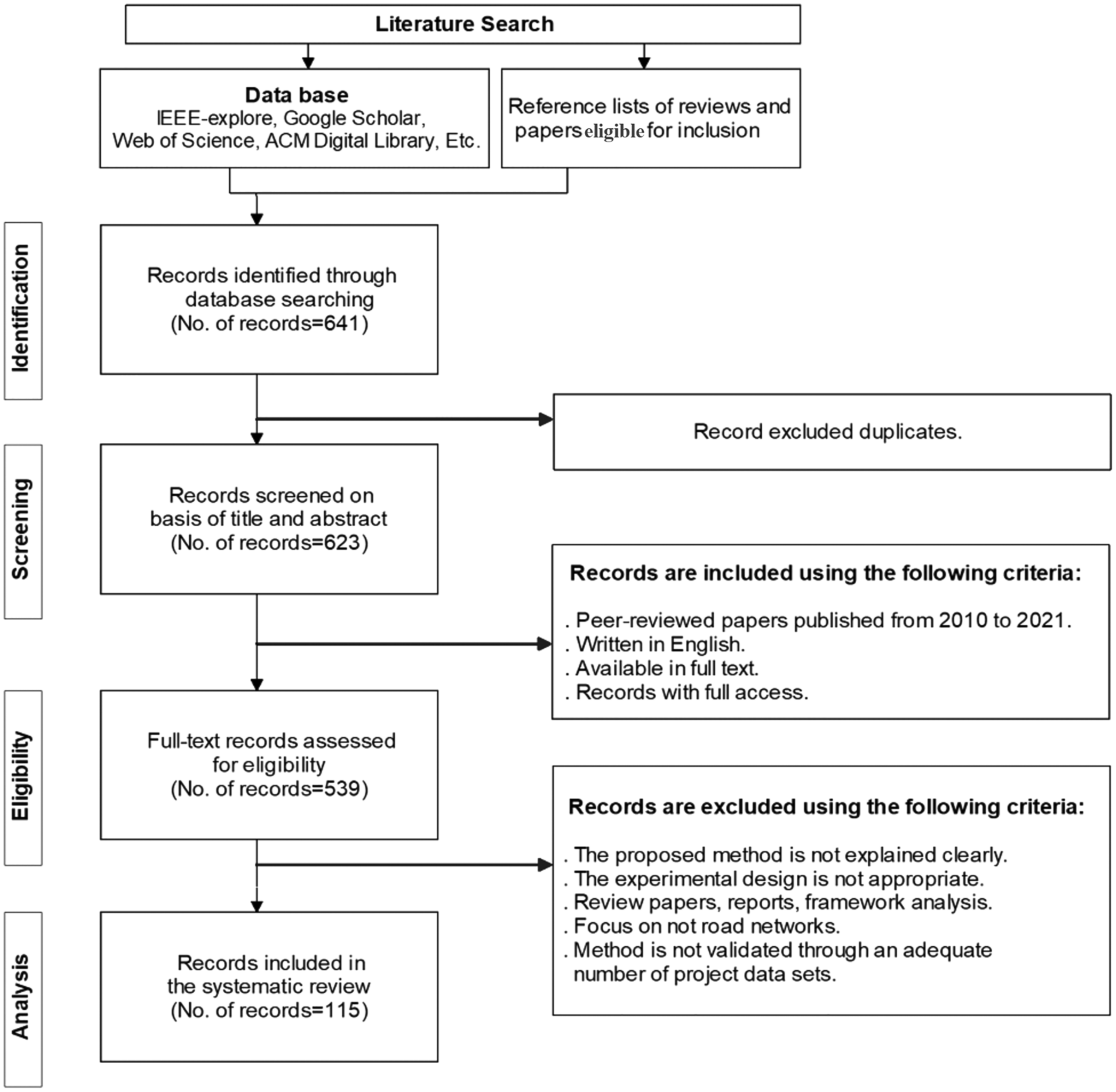
An overview of the review process.

The current section presents the selection steps and the corresponding number of studies selected after applying the eligibility criteria. There was a total of 641 papers available in full text that were downloaded for further manual selection. For each paper, the title, abstract, and full text were reviewed by adopting the exclusion criteria to determine the final samples included in the systematic review. Figure 1 shows the process of the literature selection, including identification, screening, eligibility, and analysis, as well as the number of article changes after each stage in the process. By checking the titles and abstracts, 102 papers were removed due to the reasons of duplicates, non-English, etc. Therefore, 539 papers remained for further selection. After further reading of 539 papers in full-text, 424 papers were excluded. Finally, the manual selection resulted in 115 eligible papers for the systematic review.

### Data collection and synthesis

The data synthesized in an SLR is the outcome extracted from individual research studies relevant to the research question. In other words, the main task of this step is to summarize, extract and synthesize information and evidence from the selected papers to answer the research questions.

The data extracted from each study are:

- Classification of the paper (*about Travel time, Arrival time or both*).
- General information: the author(s) of the paper, year of publication, etc.
- The method/approach/technique proposed by author(s).
- Strengths and weaknesses of each proposed method.
- Dataset, Factors, experimental setup and the results upon evaluating the proposed method’s performance.

**Figure 2:**
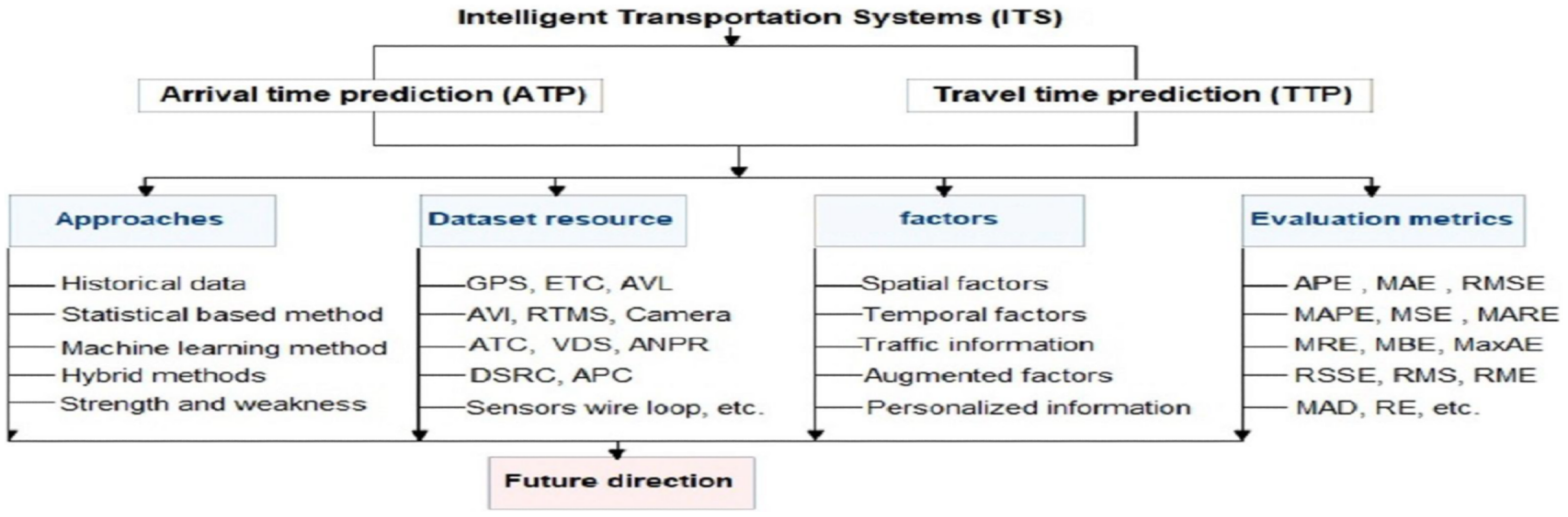
Structure of the review.

However, as shown in Fig. 2, our study is presented in four dimensions viz. approach classification, dataset resources, influence factors and evaluation metrics. The first dimension presents the classification of existing methods and their advantages and weakness. In turn, the approaches of arrival time and travel time prediction are divided into four categories: the historical data, statistical-based method, machine learning-based method and Hybrid methods. We also discuss different evaluation metrics viz. APE, MAE, RMSE, MAPE, MSE and MARE. We present various dataset resources including GPS, ETC, AVL, AVI, RTMS, ANPR, etc. Moreover, we present the factors that affect the punctuality of ATP and TTP methods and finally, we present research directions as future works.

However, Tables 1 and 2 presents the distribution of the selected papers extracted in this study after removing the duplicate and irrelevant papers. Furthermore, Tables 1 and 2 also listed some attributes of papers that are considered for the survey. These Tables include the following fields: the reference, applied techniques, the dataset resource, the evaluation metric and the influence factors. We define some terms (below), which will be used throughout the rest of the paper.

**Table 1:** Distribution of papers (TTP) based on the technique, dataset, evaluation metric and influence factor.

References	Technique	Dataset resource	Evaluation metric	Factors contributed to the proposed method
(Kumar, Mothukuri & Vanajakshi, 2021)	Kalman filter	GPS	MAPE, MAE, Mean	Day of week, Time of day, Speed, jam density
(Chiabaut & Faitout, 2021)	A Gaussian Mixture Model (Yildirimoglu & Geroliminis, 2013) & k-means algorithm	Sensors wire loop	RMSE	Time of the day, length of section, speed, congestion
(He et al., 2020)	Non-negative Matrix Factoriza- tion (NMF) (Lin, 2007), LSTM	Road Networks^1^, Bus Route^2^, Weather data^3^	MAE, RMSE, MAPE	Distance, traffic conditions, dwelling time, delays at intersections, speed, time of day, day of week, travel distance, number of bus stops, number of intersections, number of traffic signals, weather condition
(Wu et al., 2020)	the attention ConvLSTM	Traffic datasets from TfNSW	RMSE, MAE	Departure time, waiting time, running time, number of bus stops, arrival time at the station
(Chen, Liang & Chu, 2020)	Gradient boosting (Friedman, 2001)	ETC data	MAPE, MAE, RMSE	Time of the day, day of the week, national holiday, big event/activity, narrowing of roadway
(Abdollahi, Khaleghi & Yang, 2020)	K-Means, Deep stacked autoencoder, MLP	TLC, Weather	MAE	Weather, weekend/weekdays, time of day, distance and direction, average speed
(Gündüz & Acarman, 2020)	RNN-LSTM	GNSS data^4^	RMSE, MAE	Day of the week, average travel speed, traffic congestion, time of day, Position.
(Kang et al., 2020)	XGBoost	Alibaba Cloud Tianchi dataset platform	MAPE, MAE, RMSE	Average speed, day of week day of year, vacation, length (length of each road segment), width (width of each road segment), area (area of each road segment)
(Ting et al., 2020)	GRU, XGBoost	Traffic dataset of the Taiwan Freeway Bureau,	MAPE, RMSE, MAE	Average travel time, average traffic speed
(Liu et al., 2020a)	Kalman filter	RFID	MAE, RMSE, MAPE	Traffic flow diversion rate (TFDR)
(As, Mine & Yamaguchi, 2020)	ANN, SVR	GPS	MAE, RMSE	Days of the week, time of day
(Yuan et al., 2020)	RNN, DNN	GPS, AVL	MAE, RMSE, MAPE	Day of a week, distance, time of day, dwell time, speed
(Petersen, Rodrigues & Pereira, 2019)	CNN-LSTM	AVL	MAE, RMSE, MAPE	Time of D/W/M/Y, departure time, travel time, arrival time
(Wang et al., 2019)	A method based on the *ARIMA Model*	GPS	MAE MRE	Speed, distance, departure time
(Xu et al., 2019b)	ANN	GPS	RMSE, MAPE	Distance, travel time
(Xu et al., 2019a)	STDR (LSTM & CNN)	GPS	MAE, MAPE MRE	Weather, time of D/W/M/Y, zone information(work zones), position(location) of vehicle
(Shen et al., 2019)	TCL		MAE, RMSE MAPE	Speed, distance, time of D/W/M/Y personalized information, departure time, position(location) of vehicle
(Li et al., 2019)	K-means & ELM	AVI microwave radar detectors	MAPE	Weather. Accidents, speed
(Zhang & Chen, 2019)	DT & Regression	GPS sensors wire loop	–	Weather
(Zhao et al., 2018a)	GRU	RTMS DSRC	RMSE, MAPE	Travel time
(Tang et al., 2018)	TBBP	GPS	MAE, RMSE MAPE	Traffic flow, distance, personalized information, travel time
(Ran et al., 2018)	CNN	Highways England	MAE, RMSE, MAPE	Traffic flow, time of D/W/M/Y
(Cheng, Li & Chen, 2018)	GBDT	data collected in Nanjing were used.	MAPE, MAD, RE	Traffic flow, speed, travel time, density, vehicle type, occupancy
(Elhenawy, Hassan & Rakha, 2018)	Linear regressions	GPS	MAPE, MAE	Congestion, distance time of D/W/M/Y
(As & Mine, 2018)	ANN	GPS	MAPE	Time of D/W/M/Y, travel time
(Fan et al., 2018)	RF	ETC/toll systems	MAPE	Accidents, speed, distance, time of D/W/M/Y, travel time, vehicle type
(Li et al., 2018a)	FRNNM	AVI ETC/toll systems	APE, MRE, MARE	Weather, time of D/W/M/Y
(Yu et al., 2018)	RFNN	GPS	MAPE	Bus stop, speed
(Zhao et al., 2018b)	K-NN	RTMS	MAPE	Congestion, speed, time of D/W/M/Y
(Yang, Zhang & Zhang, 2018)	BGM	ETC	MAE, MAPE MRE	Speed, distance, time of D/W/M/Y Passenger demand, travel time
(Wang, Fu & Ye, 2018)	ANN, RNN, Regression	GPS	MAE, MAPE MSE	Delay time, congestion, speed distance, time of D/W/M/Y Personalized information, departure time, intersection
(Fathy & Mohammadi, 2018)	ANN	GPS sensors wire loop CAMERA	RMSE	Congestion, time of D/W/M/Y travel time
(Li et al., 2018b)	ANN	GPS	MAE, MAPE MARE	Distance, departure time, travel time
(Liu et al., 2017)	LSTM-DNN	PeMS	RMSE, MAPE	Travel time
(As, Mine & Nakamura, 2017)	ANN	GPS	MAE, MAPE	Time of D/W/M/Y, travel time
(Zhang et al., 2017a)	CNN-LSTM	Case study: expressway	MAE, MAPE	Congestion, travel time
(Woodard et al., 2017)	HMM	GPS	MAE	Speed, distance
(Li et al., 2017)	A proposed algorithm	GPS	RMSE	Congestion, travel time
(Cristóbal et al., 2017)	A methodology – based procedure	AVL		Dwell time, running time
(Zhang et al., 2017b)	Pattern-matching method	GPS	MAE, MAPE	Traffic flow, speed time of D/W/M/Y
(Ma, Al Khoury & Jin, 2017)	KF	ANPR		Delay time, travel time
(Kumar, Vanajakshi & Subramanian, 2017)	KF & K-NN	GPS	MAE, MAPE	Traffic flow, speed
(Ma et al., 2017)	Markov chain approach	GPS AVI	MAD	Speed, distance
(Zhu et al., 2016)	A model based on the ARIMA model	Sensors wire loop	MAE, MAPE, MSE	Congestion; Speed
(Ciskowski et al., 2016)	ANN (MPL)	Camera	MSE	Speed, time of D/W/M/Y, density
(Li et al., 2016)	ELM	Regiolab-Delft	MSE	Accidents, travel time
(Zhang et al., 2016)	SAE	data is offered by the South Guangzhou	RMSE, MAPE	Travel time
(Li & Bai, 2016)	SGPR		RMSE, MAPE	Speed, time of D/W/M/Y travel time
(Caceres, Hwang & He, 2016)	Probabilistic model	–	–	Weather, accidents, speed time of D/W/M/Y
(Amita, Jain & Garg, 2016)	ANN	GPS	MAPE, RMSE	Departure time, passenger demand travel time
(Derbel & Boujelbene, 2015)	DBN	sensors wire loop	MAE, RMSE MAPE	Congestion, weather, speed
(Yin et al., 2015)	GM model and the EM algorithm	Sioux Falls network	MAPE	Distance, travel time
(Zhang & Haghani, 2015)	TBE methods	GPS sensors wire loop	MAPE	Time of D/W/M/Y travel time
(Nantes et al., 2015)	PTTP	AVI	accuracy	Departure time, Travel time
(Fan & Gurmu, 2015)	HA, KF and ANN	AVL	MAPE	Time of D/W/M/Y, bus stop travel time
(Gurmu & Fan, 2014)	ANN	AVL	MAPE	Time of D/W/M/Y, travel time id number of the bus station
(Elhenawy, Chen & Rakha, 2014)	RF	GPS	MAE, MAPE	Congestion, speed
(Kormáksson et al., 2014)	LR	GPS	MAE, MAPE	Distance, time of D/W/M/Y, Departure time, travel time
(Uchida, 2014)	Statistical method a model based on the User Equilibrium (UE) method (Clark & Watling, 2005)	UE traffic	Mean travel time	Speed, travel time
(Jiménez-Meza, Arámburo-Lizárraga & de la Fuente, 2013)	HD	GPS	–	Congestion; Speed; Distance
(Zheng & Van Zuylen, 2013)	ANN	GPS	RMSE, MAPE	Speed, departure time, position(location) of vehicle
(Li & Chen, 2013)	ANN	Sensors wire loop ETC/toll systems AVI	MAPE	Congestion, weather, speed time of D/W/M/Y, Travel time
(Deng, He & Zhong, 2013)	Bayesian Network	GPS	MAE	Congestion, speed
(Zeng & Zhang, 2013)	ANN	AVI microwave radar detectors	MAPE MSE	Traffic flow, speed, occupancy
(Yuguang, Chaoyang & Qingquan, 2013)	A location-speed method	FCD	–	Speed; Distance; Intersection
(Yildirimoglu & Geroliminis, 2013)	Statistical procedure bottleneck identification algorithm (Chen, Skabardonis & Varaiya, 2004), GM Model and Stochastic congestion maps	GPS sensors wire loop	MAE, MAPE	Congestion, speed, travel time
(Baptista, Bouillet & Pompey, 2012)	K-NN	GPS	MPE	Weather, dwell time, running time
(Masiero, Casanova & de Carvalho, 2011)	SVR	GPS	MSE	Distance, time of D/W/M/Y departure time
(Shen & Huang, 2011)	TDNNs & MLP	8.25-mile, Florida	MAE, MAPE	Congestion, time of D/W/M/Y
(Kumar, Vanajakshi & Subramanian, 2011)	KF	GPSS	MAE, RMSE MAPE	Dwell time
(Myung et al., 2011)	K-NN	ATC VDS	MAE, MAPE	Delay time, departure time travel time
(Li & Rose, 2011)	ANN	AVI	MAE, RMSE MARE	Weather, time of D/W/M/Y travel time
(Liu & Wang, 2010)	LR	GPS	RMSE, RME	Distance, departure time, travel time
(Chang, Chowdhury & Lee, 2010)	NB & Rule-based classification	GPS	MARE	Speed, time of d/w/m/y, departure time
(Nath et al., 2010)	K-means	GPS	MARE	Speed, time of D/W/M/Y travel time
*Highways England	https://data.gov.uk/dataset/dc18f7d5-2669-490f-b2b5-77f27ec133ad/highways-agency-network-journey-time-and-traffic-flow-data.			
*Regiolab-Delft	http://www.regiolab-delft.nl			

**Notes:**

1 https://www.openstreetmap.org/export.

2 https://www.mytransport.sg/content/mytransport/home/dataMall.html.

3 https://www.timeanddate.com/weather/singapore/singapore.

4 Global Navigation Satellite Systems.

**Table 2:** Distribution of papers (ATP) based on the technique, dataset, evaluation metric and influence factor.

References	Technique	Dataset resource	Evaluation metric	Factors contributed to the proposed method
(Xie et al., 2021)	ConvLSTM	GPS	RMSE, MSE, MAE	Departure hours, departure minutes, Days of week, Holidays, distance, Weather
(Liu et al., 2020b)	LSTM, ANN	Xingtai bus company	MAE, RMSE MAPE	Working days, Weekends, Holidays, Speed, Distance
(Lai et al., 2020)	Wavelet neural network (WNN) model, Swarm optimization algorithm	bus line 102 in Suzhou city	RMSE	Travel time, Time period of the day, Weather conditions: (rainy day, sunny day), Days of the week
(Han et al., 2020)	LSTM	GPS	MSE, RMSE, MAE	Traffic factors, Travel factor, Dwelling factor, Stop dwelling factor, Weekday and weekend.
(Vignesh, Achar & Karthik, 2020)	Markov reward process (Bertsekas & Tsitsiklis, 1996; Sutton & Barto, 2018)	Indian city and of length 28 kms	MAPE	Travel times, Segment length between any two consecutive bus-stops, Running time, Dwell times
(Panovski & Zaharia, 2020)	Fully connected neural networks (FNN)	Data^1^ provided by the French government	MSE	TDM^2^ (*Traffic Density Matrix*)
(Sharmila, Velaga & Choudhary, 2020)	the Cox regression models, and AFT models	GPS	RMSE MAPE	Distance, speed, Bus stop, Dwell time, Passenger count, Gradient of the road, Intersection length, Signal details (included green time, red time, cycle length)
(Liu, Sun & Wang, 2020)	RNN-LSTM	GPS^3^	MAE, RMSE MAPE	Number of bus stops
(Hashi et al., 2019)	SVM, KF	AVL	RMSE	Traffic condition, Dwell time, Time period, Traffic Counts, Travel Time
(Achar et al., 2019)	Kalman filter (KF)	GPS	MAPE, MAE	Travel time
(Čelan & Lep, 2018)	An algorithm based on the current location of the bus and knowledge of a predetermined bus route trajectory	GPS	MAE, MRE	Railway crossing, Delay time Time of D/W/M/Y, Bus stop Departure time, Travel time
(Peng et al., 2018)	Genetic algorithm & SVM	Shenyang, China	MAE, RMSE, MAPE	Weather, Distance, Time of D/W/M/Y, Dwell time, Running time, Intersection
(Hua et al., 2018)	SVM, ANN, LR	AVL	MAE, RMSE, MAPE	Traffic flow, Dwell time, Travel time
(van der Spoel, Amrit & van Hillegersberg, 2017)	Adaboost.M1 (Freund, Schapire & Abe, 1999)	Questionnaire (230 truckers), Weather information (KNMI)	Accuracy	Congestion, Weather, Time of day, Incidents
(Zhang et al., 2017)	k-mean & ANN		MAE, MAPE	Time of D/W/M/Y, Dwell time Running time
(Jisha, Jyothindranath & Kumary, 2017)	KF	GPS	RMSE	Speed, Distance, Departure time Position(location) of vehicle
(Xu & Ying, 2017)	A proposed clustering algorithm	GPS	MAE, RMSE, MAPE	Speed, Distance, Time of D/W/M/Y Dwell time
(Treethidtaphat, Pattara-Atikom & Khaimook, 2017)	ANN	GPS	MAE, RMSE MAPE	Speed, Distance, Time of D/W/M/Y
(Singh, Bansal & Sofat, 2017)	ANN, KF & LR	GPS	MAE, RMSE MAPE	Congestion, Dwell time, Departure time, Travel time
(Yang et al., 2016)	Genetic algorithm & SVM	AVL	RMSE	Weather, Speed, Distance, Time of D/W/M/Y
(Padrón et al., 2016)	K-means & statistical method	AVL	--	Speed, Position(location) of vehicle
(Dhivyabharathi, Kumar & Vanajakshi, 2016)	k-NN	GPS	MAPE	Distance, Travel time
(Agafonov, Chernov & Sergeev, 2015)	Regression model	GPS	MAE, MAPE	Signals (traffic lights), Weather Distance, Time of D/W/M/Y, Density
(Agafonov & Myasnikov, 2015)	LR	GPS	MAE, MAPE	Departure time, Position(location) of vehicle
(Khetarpaul et al., 2015)	Fuzzy logic and Neural Networks	AVL GPS	RMS, MAPE	Delay time, Speed, Time of D/W/M/Y, Bus stop, Departure time, Arrival time
(Lam & Bouillet, 2015)	kernel regression (KR) method	GPS	RMSE	Arrival time
(Maiti et al., 2014)	HD model	Siruseri, Chennai	RMSE	Speed, Distance, Departure time Position(location) of vehicle
(Fu et al., 2014)	FSM	GPS		Speed, Distance, Bus stop Position(location) of vehicle
(Yu et al., 2013)	Linear regression	GPS	MAPE	Speed, Distance, Time of D/W/M/Y
(Gong, Liu & Zhang, 2013)	HMADA	GPS	MAPE	Speed, Distance, Dwell time, Departure time, Travel time
(Lin et al., 2013)	HANN model	GPS	RAE RVOR	Delay time, Time of D/W/M/Y Dwell time, Travel time
(Sinn et al., 2012)	KRP	GPS	RMSE	Departure time, Position(location) of vehicle
(Zheng, Zhang & Feng, 2012)	SVM & KF	Dalian, China	MAPE	Distance, Time of D/W/M/Y Bus stop
(Pan et al., 2012)	BP neural network	GPS	MSE	Speed, Distance, Time of D/W/M/Y Bus stop
(Biagioni et al., 2011)	HMM	GPS	APE, MAE, RMSE MAPE, MSE	Distance, Travel time
(Li et al., 2011)	LR	GPS sensors wire loop	APE MAPE MSE	Congestion, Speed, Dwell time Departure time, Intersection
(Yu, Lam & Tam, 2011)	SVM		MAE, RMSE MAPE	Speed, Arrival time
(Zhu et al., 2011)	A model-based prediction algorithm	GPS	MAPE	Speed, Distance, Bus stop, Departure time, Intersection
(Padmanaban et al., 2010)	KF	GPS	APE	Delay time, Congestion, Speed Distance, Bus stop
(Yu et al., 2010)	SVM & KF	GPS	RMSE	Weather, Time of D/W/M/Y, Travel time

**Notes:**

1 SOeS, “mars 2015 Chiffres clés du transport.”

2 The TDM technique introduces localized information about the traffic conditions in a given city area, with the help of a set of measurement stations, which capture the number of vehicles that are present in the vicinity of the considered measurement point.

3 https://data.gov.ie/dataset/dublin-bus-gps-sample-data-from-dublin-city-council-insight-project.

- Hierarchical ANN (HANN) model, Decision tree (DT), Random Forest (RF),
- Historical Data based (HD) model, Floating car data (FCD), Kalman filtering (KF)
- Random Forest (RF) (Friedman, Hastie & Tibshirani, 2001), Probabilistic travel time progression (PTTP),
- Historical Average (HA), Tensor-CNN-LSTM (TCL), Tree-based ensemble (TBE),
- Fuzzy rules neural network model (FRNNM), Deep neural network (DNN),
- Sparse Gaussian Processes Regression (SGPR), Support Vector Machine (SVM),
- Linear regression (LR) (Hosmer, Lemeshow & Sturdivant, 2013), global positioning systems (GPS),
- Extreme Learning Machine algorithm (ELM), Bayesian graphical model (BGM),
- Convolutional neural network (CNN) (Kim, 2014), Support Vector Regression (SVR),
- Tensor-based Bayesian probabilistic model (TBBP), Artificial Neural Network (ANN),
- Random forests based on the near neighbour (RFNN), Stacked automatic encoders (SAE),
- K-Nearest Neighbours (KNN) (Yang & Liu, 1999), Fuzzy rules neural network (FRNN),
- Naive Bayesian (NB) classification (Manning, Manning & Schütze, 1999), Neural network (NN),
- Kernel Regression Prediction (KRP)(based on the K-NN),
- Gradient boosting decision tree (GBDT),
- Time-delayed neural networks (TDNNs),
- Expectation–maximization (EM) algorithm (Bilmes, 1998),
- long short-term memory (LSTM) (Hochreiter & Schmidhuber, 1997),
- Bayesian network (a type of statistical model) (Derbel & Boujelbene, 2015),
- Gated recurrent units (GRU)(a gating mechanism in recurrent neural networks),
- Backpropagation (BP) neural network algorithm (Li et al., 2012),
- (PeMS) (Liu et al., 2017), an online system that provides traffic data collection over 16,000 loop detector in California the U.S.
- HMADA: a method based on hybrid “Moving average model (MAM) ” and “Moving average dynamic adjustment model (MADA)”.
- Finite State Machine (FSM) (https://medium.com/@mlbors/what-is-a-finite-state-machine-6d8dec727e2c),
- Gaussian mixture (GM) model (https://towardsdatascience.com/gaussian-mixture-models-d13a5e915c8e),
- The autoregressive integrated moving average (ARIMA) (https://people.duke.edu/~rnau/411arim.htm),
- Dynamic Bayesian Networks (DBN) (https://en.wikipedia.org/wiki/Dynamic_Bayesian_network#cite_note-5).

It is worth noting the other abbreviations will be indicated in the corresponding section.

## Analysis and Discussion

This section aims to answer the questions presented in the section “Research Questions”. In this review, we focus on methods used for TTP and ATP on road. We present a classification of various methods and the key challenges associated with each classification. Furthermore, we also summarize each paper in different aspects viz. presenting various evaluation metrics, dataset resource, factors that were taken into consideration and describing the essential concepts. We indicate future directions along with our views on the above. Moreover, in Tables 1 and 2, we included an overview of the articles selected for the systematic review. In these tables, we included the reference (*author/year*), technique (*method proposed by Author(s)*), dataset resource (*the resource of traffic data*), evaluation metric, factors contributed to the proposed method.

However, In the following sub-sections, we examined: (1) the proposed methods/techniques to model ATP and TTP on road networks (RQ (1)), in the section “*Proposed Methods for ATP and TTP*”; (2) the challenges associated with the existing techniques (RQ (2)), in section “*Strengths and Weaknesses*”; (3) the evaluation metrics, in the section “*Evaluating ATP and TTP approaches*”; dataset resources, in section “*Dataset collection systems*”, factors involved in ATP and TTP, in the section “*The factors contributing to ATP and TTP*”; (4) feasible opportunities as future direction, in the section “*Conclusion and future work*”.

### Proposed methods for ATP and TTP

This study reviews many research articles regarding the state-of-the-art in ATP and TTP. The result extracted from our review shows that the existing method can be categorized into three main groups, depending on the nature of the techniques employed, which are: *(i) Historical data-based approach*, *(ii) Statistical based approaches*, and *(iii) Machine learning-based approaches*. Also, under some conditions the hybrid approach, which combines different techniques, can be employed. In the next section, we will briefly explain the summary of all three classes and present the most commonly used methods for ATP and TTP. The results of the findings are presented in Tables 1 and 2. To be more specific, these Tables present some of the proposed methods and their main features such as the technical field that describes the technique that each method has employed. The factor field concerns the factors that have been used for TTP and ATP. The dataset resource field indicates the data collection systems. The table also contains a field about the evaluation metrics that indicate various formulas.

### A taxonomy of the ATP and TTP methods

Several research works have been conducted for ATP and TTP using a variety of techniques and approaches. However, here we discuss the most widely used approaches in what follows.

### Historical Data based method (HDM)

HDM (Kumar, Vanajakshi & Subramanian, 2017; Tang, Chen & Khattak, 2018) is employed for ATP and TTP of a particular period using the observed historical data of previous trips in the same period. Due to the easy implementation, low computational time, and filtering of the data (*decomposing data into sub-data based on the weekday, month, or other attributes*), an HDM is widely employed in practice. However, the performance of this model is generally low since an HDM is reliable when the traffic condition is assumed to remain constant over time. Therefore, any change in traffic conditions (*in the event of congestion and accidents*) affects the accuracy of the HDM. HDM employs two main methods: average travel time (ATT) and the average speed (AS) method which use historical ATT and AS of vehicles to forecast arrival time and travel time respectively. A historical data-based model usually uses the data collected by GPS technology as the distance between two points/positions is obtained using latitude and longitude of the location.

### Statistical based method

A variety of statistical-based methods for ATP and TTP have been developed. Here we describe the most widely used methods.

***Regression-based method***—the regression model is an approach that has been also used for predicting arrival/travel time. It explains a dependent variable with a set of independent variables using a linear mathematical function (Dhivyabharathi, Kumar & Vanajakshi, 2016; Khetarpaul et al., 2015). A regression-based method usually measures the impact of different factors (*independent variables/parameters*) that affect the dependent variable. ATP and TTP can be affected by different factors including intersections, traffic congestion, distance, dwell times, etc. However, the precision of a regression model depends on identifying suitable independent variables and depends on the correlation and linearity between them.

Unlike HDM, a regression-based method can work even the traffic condition is not steady. Hence, the regression model is employed by many researchers for ATP and TTP.

***Kalman filtering based method***—Kalman Filter (KF) (https://www.kalmanfilter.net/default.aspx) (Cathey & Dailey, 2003; Chien, Ding & Wei, 2002) is one of the most common prediction approaches that has been also widely used to predict arrival/travel time. It is a linear recursive predictive update approach that uses a series of measurements observed over time which may be noisy, inaccurate, and uncertain to predict the parameters of a process model. Furthermore, the basic function of the Kalman Filter is to provide a prediction of the future system state, based on the past estimations. Generally, the Kalman filter algorithm includes two main steps: prediction/propagation and update/correction. The prediction step obtains estimates of the current state variables, along with their uncertainties. The update step incorporates a new measurement to modify the propagated current state and error covariance estimates. KF has a simple form and requires small computational power. It illustrates its usefulness in numerous applications. A common application is for navigation and control of vehicles. KF is also a widely applied concept in time series analysis. The time series model (Kumar, Vanajakshi & Subramanian, 2017; Xu & Ying, 2017) can also be used to predict arrival/travel time. They use a statistical model to forecast future events based on known past events.

### Machine learning-based method

AI is a branch of computer science where the idea is to simulate the human brain. In other words, it is a method to do a task that is potentially easy for humans, but difficult for machines. In the intelligent transport systems domain, several AI applications have been developed and implemented in a variety of ways such as *autonomous vehicles* (AVs rely on deep learning algorithms). These algorithms can instruct the vehicle on how to drive in safe mode. The application of AI can be also seen in the field of *Public Transportation* (predictive models, traffic control system, etc.).

**Figure 3:**
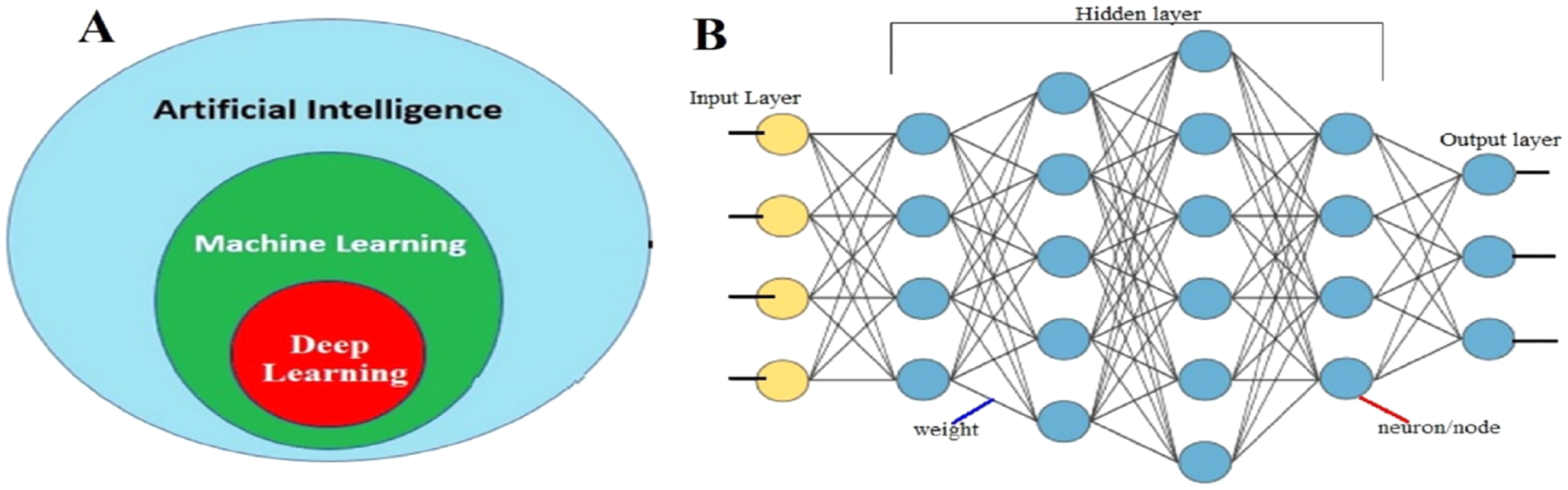
(A) Subsets of artificial intelligence; (B) deep neural network structure.

As shown in Figure 3A, Machine learning (ML) is a subclass of artificial intelligence. Its main task is to extract key features from data to cope with complex problems and handle large collections of data. An ML approach in the transportation area aims to apply known ML methods to the ITS to predict the arrival/travel time. An ML-based approach can be divided into three main classes: supervised, unsupervised and semi-supervised learning approaches. In the following sections, this classification will be briefly explained.

***Deep learning-based method—***there are several applications of traditional machine learning methods which have been proposed to predict traffic data. However, most of them have employed shallow traffic models which were considered unsatisfactory for big data scenarios (Lv et al., 2014). Hence, deep learning (DL) (https://nl.mathworks.com/discovery/deep-learning.html) based methods have attracted the attention of researchers. DL, a sub-class of machine learning, is a method to simulate the human brain. It is a NN (Figure 3B) with several hidden layers, where each layer includes a different number of nodes. Furthermore, a neural network can solve complicated problems such as non-linear relationships. The main disadvantage of this type of model is that results obtained from using this model for one location may not be transferrable to the next because of location-specific circumstances (geometry, traffic control, etc.). There are various deep learning algorithms such as deep neural network (DNN), convolution neural networks (CNN), recurrent neural network (RNN), etc.

***Supervised machine learning method*** (https://machinelearningmastery.com/supervised-and-unsupervised-machine-learning-algorithms/)—given labelled data, (training data), *D* = {(*d*_1_, *c*_1_), (*d*_2_, *c*_2_), …,  (*d*_*i*_, *c*_*i*_)}, where *d*_*i*_ is sample and *c*_*i*_ is a class name. A supervised machine learning method learns how to classify unlabelled dataset using a labelled dataset. Some commonly used supervised machine learning algorithms are SVM, NB, ANN, DT, LR, KNN and RF classifiers.

***Unsupervised machine learning method***—as mentioned before, a supervised machine learning approach needs a huge amount of labelled data for classification and its performance depends on the quality of the labelled dataset (*training and testing dataset*). However, it is time-consuming and difficult to generate labelled data and it also requires considerable human effort. With unsupervised learning methods, one can tackle the aforementioned problem. The unsupervised learning method only receives an unlabelled set of examples (**e.g*., K-Means, a calculating approach*). It is popular among many researchers since it is less dependent on the domain.

***Semi-Supervised machine learning method—***unlike the supervised and unsupervised learning methods, a semi-supervised learning method uses both labelled and unlabelled dataset. Therefore, it can be used to enhance the results compared to supervised and unsupervised learning, especially if there is limited labelled data.

### Hybrid methods

A hybrid method usually combines two or more approaches to predict arrival/travel time. For instance, Nguyen et al. (2018) and Kumar, Vanajakshi & Subramanian (2017) used a combination of SVM & KF, and KF & K-NN approaches respectively.

### Travel time prediction methods

**Figure 4:**
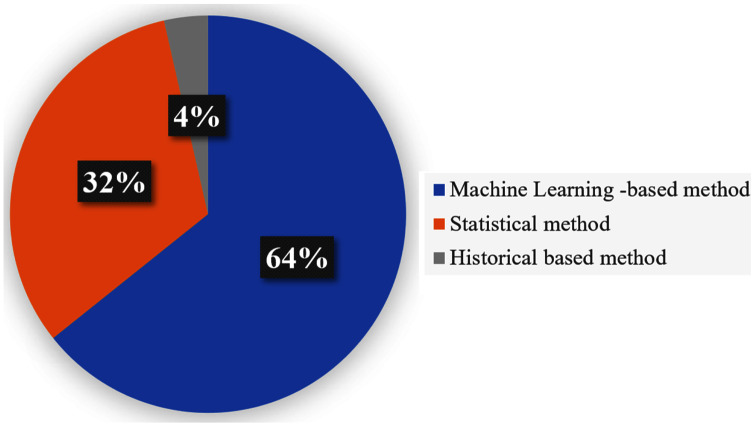
TTP approaches with frequencies.

**Figure 5:**
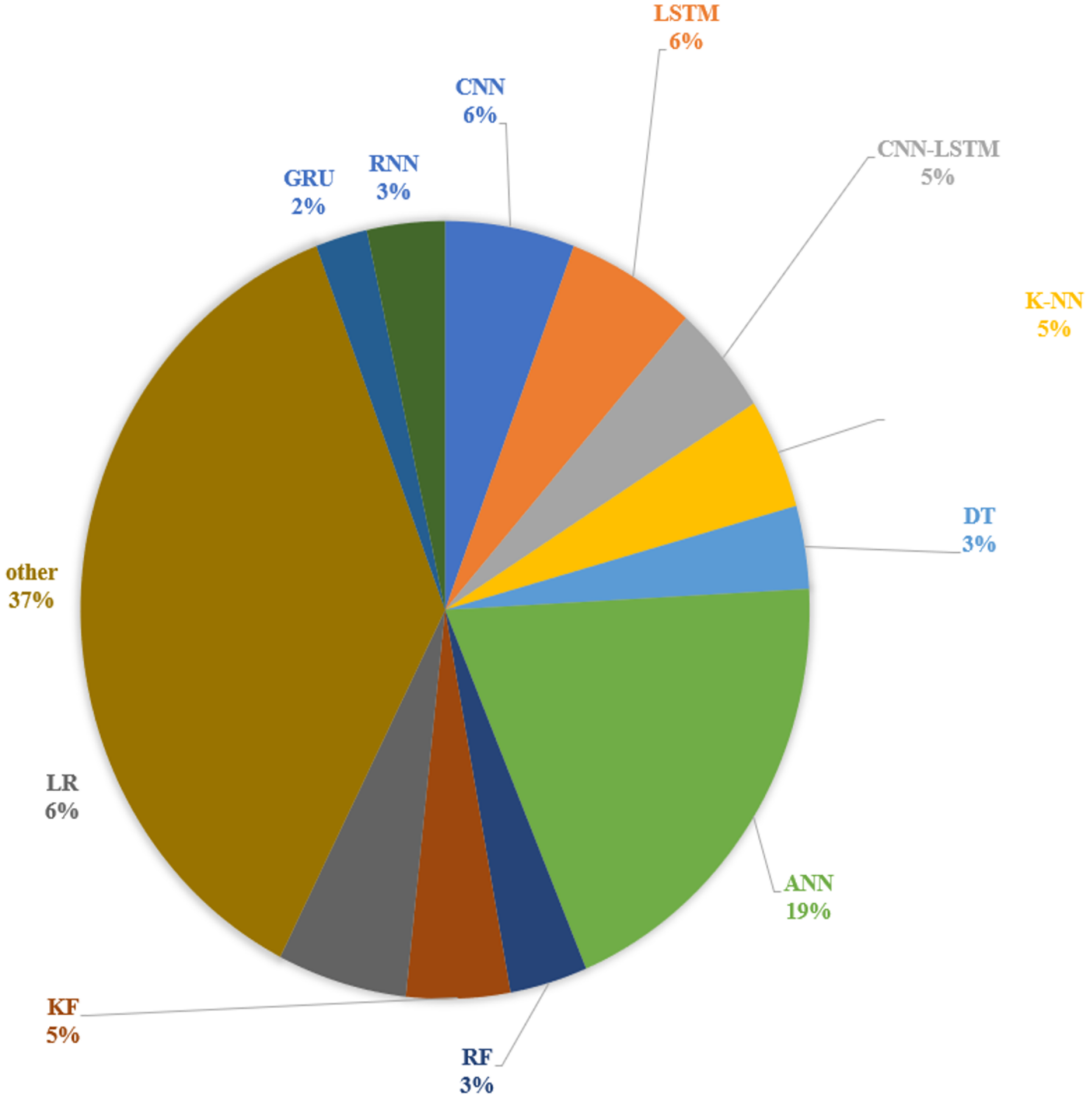
Most commonly used techniques for TTP.

We identified several TTP techniques in 75 different papers as presented in Table 1. The current review also reports the frequency at which the techniques are used based on the three categories as indicated above. In this regard, Fig. 4 shows the frequency use of Historical data-based technique, Machine learning-based technique, Statistical based technique in the travel time prediction methods. Considering the result obtained, 64% of the total papers on travel time prediction relied on a machine learning method. On the other hand, the historical data-based technique and Statistical based technique were used in 4% and 32% of the papers respectively. Therefore, results show that the machine learning-based technique can be recognized as the most commonly used approach for TTP among the existing research. This is because the machine learning method can (i) exhibit higher performance than other approaches, (ii) obtain remarkable results and also to be more effective in travel time prediction, (iii) review a large amount of data and discover specific patterns that would not be clear to humans, (iv) handle multi-dimensional data of multiple varieties, even in a dynamic or uncertain environment, (v) perform fast processing and real-time predictions. Furthermore, we elaborate more on the techniques that are commonly used and vice versa. We determined various techniques that were used in multiple articles in Fig. 5, along with their corresponding frequencies. The other techniques not mentioned in Fig. 5 were only used once for ATP in our review. As shown in Fig. 5, the most used technique is the ANN model (19%). Furthermore, K-NN (5%) and KF (5%) were also used usually, while the use of the RF model (3%), GRU (2%) and DT model (3%) seem to be stable during the past years. Additionally, CNN (6%), LSTM (6%) and LR (6%) are competing algorithms, used to a similar extent for TTP, while other papers used each of the remaining techniques for TTP (37%).

The results reveal that ANN can be considered as the most used technique for travel time prediction among researchers. Its usage is more than double that of the second most frequently used technique. This is because an ANN can perform competitively and can model non-linear and complex relationships (*relationships between inputs and outputs variables*). It also does not have any restriction on the number of input variables.

Moreover, the researchers preferred to integrate various methods to deal with the uncertainty, bring together various features/advantages of different techniques and combining various aspects of travel time. On the other hand, It has been shown that some approaches are better suited for representing some aspects of travel time prediction. For example, the RNN and LSTMs are a nonlinear time-series model (where each sample can be assumed to be dependent on previous ones). Also, the LSTM is shown to be insensitive to gap length. On the contrary, an ANN cannot model the sequence of data (each sample is assumed to be independent of the previous and row of data). Therefore, some hybrid models combine machine learning methods with statistical-based methods, or they combine Historical data-based methods with statistical-based methods. For example, Fig. 5 shows that the most used combination of CNN technique is with the LSTM technique (5%).

### Arrival time prediction methods

**Figure 6:**
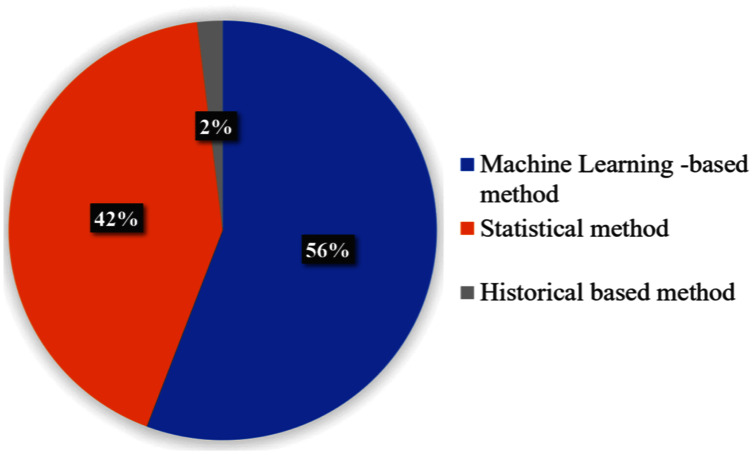
ATP approaches with the percentage.

**Figure 7:**
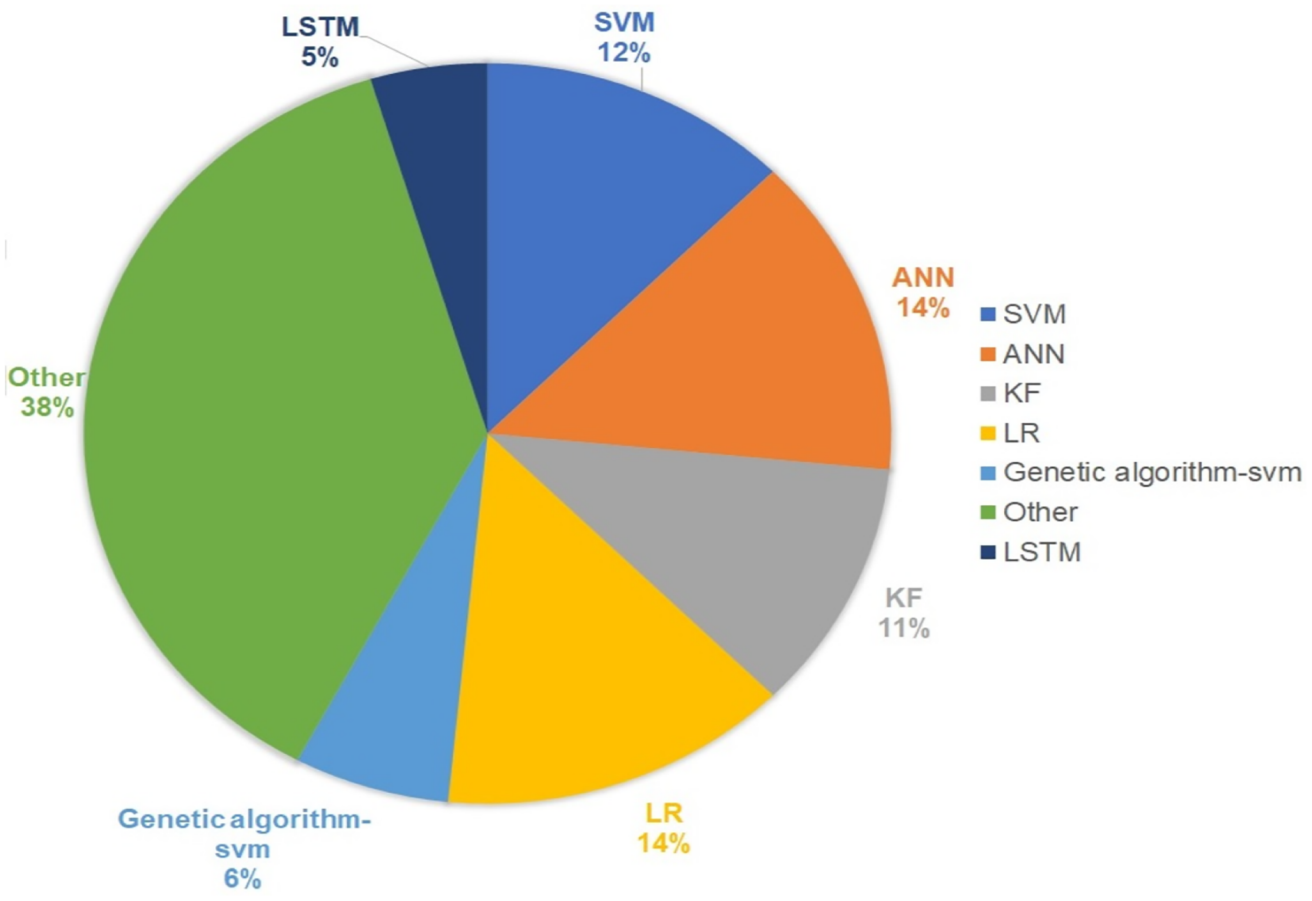
Most commonly used techniques for ATP.

The result presented in this section is about techniques used for arrival time prediction. We present the proposed techniques in Table 2. Figure 6 presents the distributions of the approaches, used for ATP techniques. We see that the three categorized approaches, namely, statistical methods, historical-based methods and machine learning-based methods were applied with frequencies of 56%, 2% and 42% respectively. The result presented in Fig. 7 shows that LR (14%) and ANN (14%) are the most used techniques. Furthermore, we see that KF (11%) and SVM (12%) are competing techniques for ATP. Also, LSTM (5%) was used sparingly, while the remaining techniques with 38% frequencies were only used once for the ATP. Some hybrid models also combine different approaches. As shown in Fig. 7, the most commonly used combination of genetic algorithm is with the SVM technique (6%).

### Strengths and weaknesses

As discussed earlier, various prediction methods were proposed for ATP and TTP using different dataset resources (**e.g*., AVL, APC, etc*.) in the last few decades. These methods can be also divided into historical data-based approaches (naive approaches), parametric method (*e.g*., LR models, ARIMA based method, KF models) and non-parametric methods (*e.g*., SVM, ANN). In this section, we discuss the strengths and limitations of each of the aforementioned groups.

***Historical data-based model***—Is fast (*low computational effort*) and easy to implement (Kumar, Vanajakshi & Subramanian, 2017). Therefore, it is widely employed in practical applications. However, the accuracy is generally low because the result that this method produces is reliable only if the traffic situation/pattern is stable in the area involved since any change in traffic conditions (road congestions and accidents) can affect the performance of the method (Khetarpaul et al., 2015). In other words, this model relies on the similarity in the traffic conditions between real-time traffic and historical traffic patterns.

***Parametric models***—In this type of model the ensemble of parameters used to specify the structure of a mathematical function (Eq. (1)) is predefined and also the dimensional space is finite. Furthermore, the relation between input variables and the target variable (output) must be defined and the parameters need to be determined carefully. Determining such a function is difficult and when the dimension space becomes larger, it becomes difficult to model the relationship between the independent variables and the dependent variable (target) because variables in transportation systems can be highly intercorrelated (Gurmu & Fan, 2014).

The most popular parametric model is linear regression (Du, Peeta & Kim, 2012) and the target variable (*i.e.*, arrival time) is a function of the various variables.

(1) $$f\left( t \right)\; = \; {\alpha _1}\; + \; {\alpha _2}{x_2}\; + \; {\alpha _3}{x_3}\; + \cdot\; \cdot\; \cdot + {\alpha _n}{x_n}$$

where, ${x_n}$ is an input variable that can be the day of the week, the weather or traffic observations. ${\alpha _n}$ is a parameter that can be determined using different techniques. Time series models (Hofleitner, Herring & Bayen, 2012; Yildirimoglu & Ozbay, 2012) and Bayesian nets (Castillo et al., 2011) are other types of model that can be also considered as parametric models,

Regression and time-series models are more accurate for the short-term compared to long-term prediction. They are also more suitable for use in free-flow rather than congested traffic and they fail to predict when an accident has occurred (Guin & Laval, 2013). Unlike historical data-based prediction methods, which can work even when the traffic conditions are not steady, both regression and time-series models find modelling nonlinear relation difficult. A time series based method (TSbM) includes the following advantages: high computation speed; speed and ease of use; simple formulation of the method; a relatively small number of operating variables (Du, Peeta & Kim, 2012; Yu et al., 2010). A TSbM assumes that all external factors of the system are constant. In other words, variation in historical data patterns or dissimilarity between real-time data and historical data can lead to wrong results (Khetarpaul et al., 2015). Since a regression model relies on historical data when facing fluctuating data, the prediction may have low precision. In such a situation one can employ another type of parametric model, namely, a Kalman filtering model. A Kalman filtering model can adapt to fluctuating traffic data with time-dependent parameters. Therefore, it is suitable for real-time applications (Khetarpaul et al., 2015; Treethidtaphat, Pattara-Atikom & Khaimook, 2017). However, since a traffic system is non-linear and complex, a Kalman filter may not always be a good option to implement.

***Non-parametric models***—Unlike parametric models the structure of the non-parametric model or the corresponding mathematical function is not predetermined. Therefore, the model can review a huge amount of data and discover specific patterns that would not be apparent to humans. Also, it can handle data that are multi-dimensional and can also do this in dynamic or uncertain environments. There are several methods for non-parametric regression such as neural networks (Li & Rose, 2011), Decision trees (Nikovski et al., 2005), SVR (Yildirimoglu & Ozbay, 2012). A non-parametric model can (i) obtain stable and robust prediction with the lowest prediction error, (ii) be able to capture the complex non-linear relationship between the target variable and the independent variables. In other words, this type of method is useful when it is difficult or even impossible to mathematically formulate the relationship between the input and output (Altinkaya & Zontul, 2013), (iii) process complicated, and noisy data, and (iv) work even if traffic condition is not steady (Comi et al., 2017).

However, non-parametric methods need a large amount of data for training (Dhivyabharathi, Kumar & Vanajakshi, 2016). Also, training them can be time-consuming. Furthermore, the result obtained using these methods for a location is generally not transferrable to the next, due to location-specific situation (geometry, traffic control, etc.). There are also general limitations to artificial intelligence (AI)-based method including (i) the relationship between the input and the output, which is determined without any knowledge to the internal computations of the system (black box), (ii) high cost for developing a smart technology due to its complexity, (iii) the computation complexity of AI-based methods (Abduljabbar et al., 2019), and (iv) the massive resources (hardware) needed to function.

Nevertheless, if algorithmic precision is important, if the computation cost is not an issue and a huge amount of data is available, then a non-parametric model could be a good option. A non-parametric model is complex, requiring a huge amount of data with noisy and nonlinear variable relationships (Treethidtaphat, Pattara-Atikom & Khaimook, 2017).

## The Factors Contributing to ATP and TTP

As we discussed earlier, ITS can be used to cope with traffic congestion, improve the efficiency of the transportation infrastructure. On the other hand, both ATP and TTP are two main aspects of ITS since they can provide useful information, enhance transit service level, etc. For this purpose, many researchers have proposed several methods for travel/arrival time prediction. The performance of these methods depends on various factors (Dhivyabharathi, Kumar & Vanajakshi, 2016; Ma et al., 2019; Zhu et al., 2011) such as the occurrence of accidents, weather condition, road construction, human factors, etc. To enhance the accuracy of ATP/TTP on road networks, various factors should be considered as indicated in Tables 1 and 2 (please see the column “*Factors contributed to the proposed method*”). Thus, the current section demonstrates the factors that influence transportation problems. Here, we need to provide a description of the basic terms/concepts that will be used throughout this section.

The factors affecting the ATP/TTP can be divided into five categories (Chang, 2005; Li & Chen, 2013; Wang, Fu & Ye, 2018) including temporal factors, traffic information, spatial factors, augmented factors and personalized information.

***Spatial factors***—The arrival/travel time is correlated with the route and environment where the travel takes place. Thus, we indicate a set of possible factors (or features) based on spatial information. The spatial factors can be the road segment, intersection, road/route length/width and the number of lanes in the segment, road construction, number of intermediate stops and turning movements, which are defined as follows:

***(a)****Road classifications*: there are two road types (freeway and urban road). Freeway is a wide road for fast-moving traffic, high-speed vehicular traffic, or an expressway with fully controlled access without any traffic signals, stop signs or railroad crossings, intersections and pedestrian paths. The urban street is the basic unit of urban space which refers to urban arterials and connectors.

***(b)****Pedestrian and cyclists crossing streets*: a pedestrian crossing is a place to cross a road/street, while bicycle crossing is a place that a bicyclist is expected to use for crossing.

***(c)****Parking space*: is an area in a street to park a car. The parking not only reduces the capacity of road facilities but also disrupts the traffic flow (vehicles moving into or out of the parking area).

***(d)****Railway crossing*: the junction between road and railway transport.

***(e)****Work zone*: a road may include different areas for instance, residential, industrial, school, etc.

***(f)****Road intersection*: refers to the junction where two or more roads/streets/lanes cross or come together at a single location.

***(g)****Urban link*: is a section/part of a street between two consecutive intersections.

***(h)****Urban segment*: is an integration of a link and intersection. Urban street/route includes several contiguous segments.

***Temporal factors***—We indicate the temporal information with various factors such as departure time, running time, time of year/month/week etc., which are defined as follows.

***(a)****Running time*: the time spent travelling between two consecutive stops/stations. Running time depends on the following features (Comi et al., 2017): (i) flow speed, which is related to link capacity/flow, and (ii) link characteristic, which is related to the number of link lanes, signals, the road work, weather conditions, etc.

***(b)****Year/month/week factors*: time variation for ATP or TTP of a route can be considered from different viewpoints (Ma et al., 2016) including, *hours in a day* that specifies the time of day which can be divided into several periods (*e.g*., Morning, Midday, Afternoon, Evening, Night), the time variation of peak and non-peak hours (Ma et al., 2017), and day of the week: which specifies the difference between the operation of working days and weekend. Furthermore, the factors like holidays (especially school holidays), seasonal variations and month of the year can be also considered.

***(c)****Departure time*: the time at which a vehicle is scheduled to depart from a point. In other words, departure time is properly specified only when a vehicle is moving or has moved in the past.

***(d)****Travel time*: refers to the time the trip takes from the origin to the destination point within a road transportation network (Amita, Jain & Garg, 2016). In order words, it is the duration of time that a vehicle takes from the start of travel to the end of travel. Travel time can be affected by various factors such as incidents, traffic lights, dwelling times, seasonal variations, route length, number of intermediate stops, etc.

The traffic information can be affected by the traffic signal. In most instances, a vehicle decelerates when it reaches a stop line or when a traffic light turns red. The vehicle stays until the traffic light turns green and then the vehicle accelerates to pass the intersection. The definition of some factors listed above is further explained as follows:

***(e)****Stop delay*: the duration for which a vehicle stands and waits for the green traffic light at the signalized intersections.

***(f)****The declaration time*: the time duration it takes for a vehicle to go from moving to standing still at the signalized intersection or stop line.

***(g)****The acceleration time*: the time duration that a vehicle takes to speed up from standing to running speed.

***(h)****Dwell time*: is the time spent at each stop/station to allow passengers to board and alight. It is therefore the time a vehicle takes for serving passengers. The dwell time depends on the number of passengers getting on and off, bus characteristic (number of doors and fare payment method), stop spacing, crowding, vehicle type, time of day, weather condition and passenger behaviour (Tirachini, 2013; Zhang & Teng, 2013). Among those factors, the passenger demand factor is the principal determinant of dwell time, and this was used by most of the studies. Dwell time can be determined by the difference between the arrival time and the departure time at the stop/station.

***(i)****Arrival time*: is defined as the time of arrival of the bus at the stop/station. Arrival time can be influenced by several factors such as driver behaviour, intersections, traffic light, dwell time (time spend in the same position), location, speed, road condition, weather condition, distance and travel time (Amita, Jain & Garg, 2016; Čelan & Lep, 2018).

***(j)****Delay time*: considers the delay (*time duration***)** of a vehicle at a stop/location. In other words, it refers to the difference between the scheduled time (based on *an external timetable which includes the scheduled time of arrival at location*) of arrival and the actual timestamp (Sinn et al., 2012). Delays are often due to weather conditions, busy roads, or a large number of passengers.

***Traffic information***—provides a picture of the current traffic situation on the road. The traffic information in the traffic network has a direct impact on ATP and TTP. The traffic information can be speed, volume, traffic incidents, etc. These are defined as follows:

***(a)****Speed*: is the distance travelled per unit of time.

***(b)****Traffic flow*: refers to the number of vehicles passing a reference point (for a given route length) per unit of time (vehicles/hour). Usually, it is affected by several factors such as road conditions, weather conditions, driving style of vehicles, number of vehicles on the road, traffic lights, time of travel. Traffic volume is also expressed as the number of vehicles crossing a point of road per unit of time.

***(c)****Event*: indicates an accident or other events that prevent the movement of vehicles.

***(e)****Traffic density*: is expressed as the number of vehicles per unit length or the number of vehicles per unit distance.

***(f)****Occupancy*: is defined as the percentage of time for which a point on the road is occupied by vehicles.

***(g)****Traffic congestion*: is a condition of transport that is determined by slower speed, longer travel times, and long vehicular queueing. It may result from an accident (one of the key sources of non-recurrent congestion (Hall, 1993; Skabardonis, Varaiya & Petty, 2003; Systematics, 2005)) on the road(s) possibly resulting in closure. Traffic congestion is also known as a traffic jam. Traffic status varies under different condition such as weather conditions or day of the weak (it depends on whether it is a weekday, weekend, public holiday and/or school holiday) (Chung, 2012; Park, Messer & Urbanik, 1998).

***Driving style***—is defined as the different driving preference for different persons (Rahman, Wirasinghe & Kattan, 2018). Driver characteristics also contribute to the variation in travel/arrival time. For instance, according to research from (Hirose, Oguchi & Sawada, 2004), a driver can be categorized into three models: “normal”, “rude” and “slow”. A rude driver reacts quickly without considering any possibility that his actions may affect traffic conditions. A normal driver reacts normally during his/her driving process, while a slow driver reacts slowly during his/her driving process. Usually, given the same zone, a rude driver will arrive earlier than a normal/slow driver. However, different characteristics of a driver can affect the ATP/TTP.

***Augmented features***—there is some information that can be employed as augmented features such as the weather information, government policies temperature and schedule adherence, which are defined as follows.

***(a)****Weather condition*: refers to the meteorological conditions along the road. The weather types can be fog, rainy, storm, snow, cloud lighting, dry, windy, sunny are dry, visibility.

***(b)****Schedule adherence*: is the difference between the scheduled time and actual arrival time (Jeong, 2005). The positive and negative results of schedule adherence indicate that the vehicle arrives late or early respectively. Indeed, schedule adherence controls the behaviour of bus drivers, and can also affect the dwell time at stops as well as the travel time.

**Figure 8:**
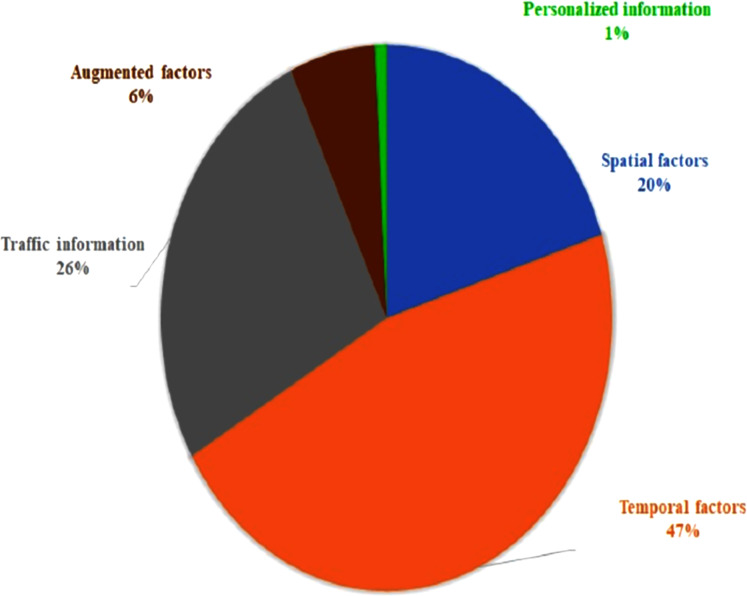
Distribution of factors used in ATP and TTP.

**Figure 9:**
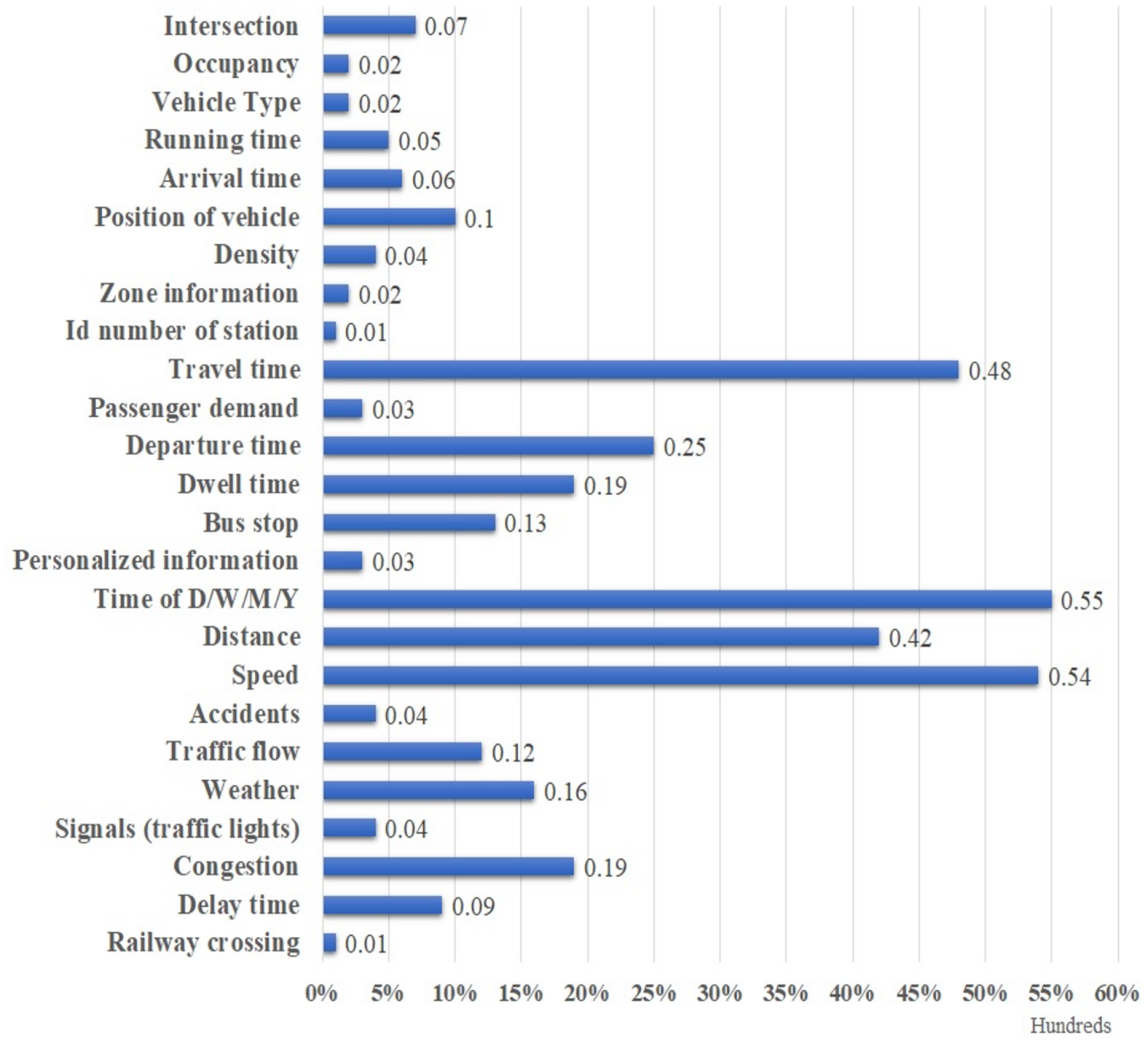
Most commonly used factors for ATP and TTP.

We also report the rate at which the factors are used based on the five main groups of the factors: Temporal Factors (TF), Traffic Information (TrI), Spatial Factors (SF), Augmented Factors (AF) and Personalized Information (PI). Figure 8 shows that the frequency use of TF, TrI, SF, AF and PI in the ATP and TTP techniques is 47%, 26%, 20%, 6% and 1% respectively. TF can be recognized as the most used factor for ATP and TTP in the existing literature. Furthermore, as shown in Fig. 9, the travel time, departure time, dwell time, bus stop, time of D/W/M/Y, distance, speed, weather, and congestion are the most frequent factors used in ATP and TTP models.

In Tables 3–5 the possible combinations that have been applied to a variety of factors are present: Table 3 indicates the combination of factors that have been used to predict travel time, Table 4 presents the combination of factors that have been employed to predict arrival time and Table 5 presents the combination of factors for both ATP and TTP.

**Table 3:** Tendencies on blended influence factors, TTP.

	Spatial factors (%)	Temporal factors (%)	Traffic information (%)	Augmented factors (%)	Personalized information (%)
Spatial factors		26.15	22.72	4.93	4.73
Temporal factors	27.75		40.26	14.45	4.73
Traffic information	24.3	41.95		12.87	4.73
Augmented factors	4.93	16.03	11.28		0
Personalized information	4.73	4.73	4.73	0	

**Table 4:** Tendencies on blended influence factors, ATP.

	Spatial factors (%)	Temporal factors (%)	Traffic information (%)	Augmented factors (%)	Personalized information (%)
Spatial factors		63.73	46.86	10.2	0
Temporal factors	57.06		44.13	6.96	0
Traffic information	50.02	53.73		6.76	0
Augmented factors	10.2	6.96	3.63		0
Personalized information	0	0	0	0	

**Table 5:** Tendencies on blended influence factors, ATP and TTP.

	Spatial factors (%)	Temporal factors (%)	Traffic information (%)	Augmented factors (%)	Personalized information (%)
Spatial factors		37.14	26.16	6.18	3.84
Temporal factors	38.00		41.40	11.22	3.84
Traffic information	25.09	41.40		8.14	3.84
Augmented factors	6.63	14.20	9.80		0.00
Personalized information	3.80	3.78	3.81	0.00	

For instance, in Table 4 the first row indicates the fact that the most used combination of spatial factors is with temporal factors (63.73%), while no one has used compound factors which brings together spatial factors with personalized information. Hence, it can be also considered as future work. Moreover, as shown in Table 3 a frequent combination of a spatial factor is with traffic information factor. Also, some researchers have tied to combine spatial factor with augmented factors or personalized information. However, a spatial factor usually is combined with a traffic information factor or with a temporal factor. It has to be referred that some researchers also combined more than two types of factors (*e.g*., TF, SF, TrI), but they are few such as TRI, TI, AI (3.98%), TRI, TI, SI (23.93%), TRI, SI, TI, AI (4.07%), TI, TRI, SI, PI (3.22%), AI,TI, SI (2.71%), SI, AI,TRI (13.29%).

## Dataset Collection Systems

Due to the progress in advanced technologies (*e.g*., Automatic Passenger Collection (APC), Automatic Vehicle Location (AVL) and Automatic Vehicle Identification (AVI), etc.) for transportation, an intelligent transportation system can extract traffic information from several sources and in different formats. These technologies are considered as the key components of intelligent transportation systems (ITS), as well as what can be used to provide more accurate arrival/travel time information for transportation management. Data collection systems (to collect traffic data) are categorized into two main groups: (i) station-based/fixed sensors/point detectors/spot collection methods and, (ii) mobile sensors/ point-to-point detectors/mobile detectors/spatial collection methods/Active Fixed Detectors.

The more detailed information about the most common data collection systems is as follows.

***(a)****Fixed point data collection system*—the current system is installed in a fixed point on a road. It also collects traffic information in this specific spot and consists of loop detectors. A loop detector can either be a single or a double loop detector (Chu, Oh & Recker, 2005; Van Lint & van der Zijpp, 2003; Yeon, Elefteriadou & Lawphongpanich, 2008). When a vehicle crosses a loop detector, the metal mass of the vehicle causes a change in the magnetic flux in the loop producing an impulse in the current through the loop; these impulses can be used to count cars. There are other technologies used for fixed a point such as video cameras, microwave, infrared, radar, Remote Transportation Microwave Sensors (RTMS).

***(b)****Point to point data collection system*—unlike the fixed-point data collection system that can capture traffic information from a single point, a point-to-point system can capture traffic information of a vehicle between two points. It includes Automatic Toll Collection systems (ATC), global positioning systems (GPS) equipped vehicles, mobile devices, Bluetooth sensors, Automatic Vehicle Location (AVL) systems, Automatic Vehicle Identification (AVI) system, Dedicated Short-Range Communications (DSRC) and license plate number (LPN) system (that includes gathering the vehicle LPN, matching the license plate between two points and noting the arrival time at various points).

***(c)****AVL system*—is used to track the position of a vehicle at a specific point in a road using the GPS receiver. AVL is also called Automatic Vehicle Monitoring (AVM) or Automatic Vehicle Location and Control (AVLC) systems. An AVL system provides the following information: vehicle ID, the location of the vehicle (latitude, longitude), receiving time, current speed, average speed. The AVL system includes two main technologies: location and data transmission technology. Location technology is employed to calculate the real-time position of each vehicle, while data transmission relays the information to a central location. The application of an AVL system has some advantages such as being able to collect real-time information, enhancing schedule reliability, reducing operating costs, enhancing service efficiency and safety.

***(d)****GPS*—is a satellite-based navigation system that is controlled by the U.S. and used in military, ships, aircraft and civilian applications worldwide. The GPS includes 24 satellites that transfer the traffic information to GPS receivers. To calculate the traffic variable (*e.g*., velocity, current time), the signals from at least four satellites are used. Furthermore, the GPS has two positioning services: a precise positioning service that is used by U.S (military) and a standard positioning service that is employed by civilian users worldwide. However, the GPS provides higher accuracy, consistency, automation, and easier integration between collected data and GIS-based data. A geographic information system (GIS) is a system to collect, record, analyse, manage, and demonstrate geographic data. GPS data includes the location of the vehicle, direction, timestamp, vehicle type and vehicle ID. The dataset collected using GPS can be analysed and it may be sent back to a mobile device or a control system for creating a graphical representation.

***(e)****Automatic Passenger Collection (APC) system*—is based on the various types of detectors including infrared light (Chen et al., 2008; Kostakos, Camacho & Mantero, 2010; Lindholm, 1977; Shahbaz & Frielinghaus, 1985) to check the presence of passengers in a vehicle and provide the information needed to improve transportation services. These detectors are installed at the entry and exit points of the vehicle. In other words, the detectors count the passengers boarding and alighting. The information that can be collected from APC includes door opening time, door closing time, travel time between two stops, number of passengers boarding at a stop, number of passengers alighting at a stop, the distance between two stops, a unique number of each stop, date of service, time of day, day of week and date of the service.

***(f)****Automatic Vehicle Identification (AVI) system*—all types of vehicles can use AVI systems. AVI is an identification technique that can be used by any moving object. I can identify vehicles in different traffic situations in a reliable way. AVI systems are used in various applications such as automatic toll collection, access control (*e.g*., parking facilities) and speed control, truck management, traffic monitoring. There are different types of AVI systems such as Radio frequency identification (RFID) and Automatic Number Plate Recognition (ANPR). In the REID system, the AVI uses RFID as the vehicle’s signature. It works base on the electronic tag which is electronically encoded with a unique identification number attached to the vehicle. When a vehicle crosses the road, the roadside antennas sends the radio frequency signals and receives the signals from the tags. In this case, all types of car information sent by the vehicle tag are read and transmitted to the central data processing unit for various purposes. On the other hand, the Automatic Number Plate Recognition (ANPR) system takes a picture of the license plate number of the vehicle and then employs then an Optical character recognition (OCR) tool to determine the characters that can be used to identify a vehicle. Apart from these technologies, Bluetooth (Bhaskar & Chung, 2013) and WIFI (Abbott-Jard, Shah & Bhaskar, 2013) based detection systems have also been used.

***(g)****Remote Transportation Microwave Sensor (RTMS)—*is a fixed-point detector based on digital radar wave technology that collects traffic information including volume, lane ID, speed, vehicle type, etc. The RTMS has some advantages such as high detection accuracy. In addition to video surveillance, the RTMS can also be used to monitor highway and urban roads for traffic information.

***(h)****Dedicated Short-Range Communications (DSRC)—*is a new type of AVI detector, which is similar to the license plate number detector (ANPR). The DSRC system can detect the information of an Electronic Toll Collection (ETC) vehicle using the installed RSU (Road-Side Unit) devices on the highway. Electronic toll collection (ETC) is a system that collects toll payments electronically.

***(i)****Camera—*various types of cameras are used to measure different aspects of the traffic such as surveillance cameras: to capture video sequences; video detection cameras: to measure traffic flow; ARTR cameras: to identify vehicle number plates; VIM cameras. These cameras store the following data: camera id, detection time, speed, vehicle type and lane number.

***(j)****Automatic Vehicle Classification (AVC) system—*is an important component of the intelligent toll collection system. It is employed to count and automatically classify vehicles entering and leaving the lane near a toll booth.

**Figure 10:**
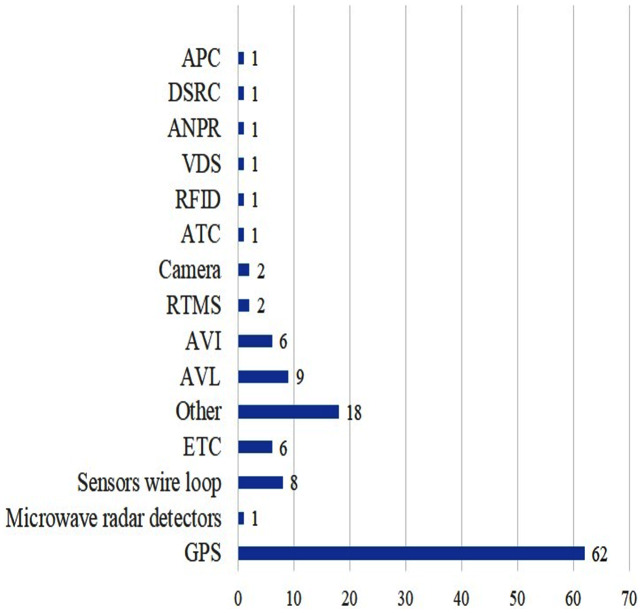
Distribution of articles based on dataset collection systems.

To summarise, in this section, we have presented different data collection systems. Figure 10 shows the overall distribution of dataset resources, divided into various categories and hence we have conducted the analysis accordingly. Most of the research has focused on the GPS, Sensor wire loop, ETC, AVL and AVI dataset systems while not many researchers explored the APC, ANPR, ATC, VDS, RTMS, DSRC, Microwave radar detectors and Camera dataset resources.

## Evaluating ATP and TTP Approaches

In this section, we discuss the evaluation metrics that are used for ATP and TTP approaches. According to the literature, there are many approaches for APT and TTP, but it is difficult to decide which method performs better based on the performance measures (shown in Table 6). This is because of the different datasets with different domain constraints.

**Table 6:** Performance metrics.

Abbreviation	Stands for	Formula
${MBE}$	Mean Bias Error	$MBE = \displaystyle{1 \over N}\mathop \sum \limits_{i = 1}^N {\varepsilon _i}$
${MaxAE}$	Maximum Absolute Error	$MaxAE = {}_{i\; \in 1:N}^{max}\left| {{\varepsilon _i}} \right|$
${MAPE}$	Mean Absolute Percentage Error	$MAPE = \displaystyle{1 \over N}\mathop \sum \limits_{i = 1}^N \left| {\displaystyle{{{\varepsilon _i}} \over {{y_i}}}} \right|100$
${RMSE}$	Root scMean Squared Error	$RMSE = \sqrt {\displaystyle{1 \over N}\mathop \sum \limits_{i = 1}^N \varepsilon _i^2}$
${RSSE}$	Root Sum Squared Error	$RSSE = \sqrt {\mathop \sum \limits_{i = 1}^N \varepsilon _i^2}$
MAE	Mean Absolute Error	$MAE = \displaystyle{{\sum \left| {{{\hat y}_i} - {y_i}} \right|} \over N}$
${MARE}$	Mean Absolute Relative Error	$MARE = \displaystyle{1 \over N}\mathop \sum \limits_{i = 1}^N \left| {\displaystyle{{{y_i} - {y_i}} \over {{d_i}}}} \right| \times 100$
${MSE}$	Mean Squared Error	$MSE = \displaystyle{1 \over N}\mathop \sum \limits_{i = 1}^N {\left( {{y_i} - {y_i}} \right)^2}$
${SSE}$	Sum Squared Error	$SSE = \mathop \sum \limits_{i = 1}^N {\left( {{y_i} - {y_i}} \right)^2}$
${APE}$	Absolute Percentage Error	$APE = \mathop \sum \limits_{i = 1}^N \left| {\displaystyle{{{\varepsilon _i}} \over {{y_i}}}} \right| \times 100$
${MRE}$	Mean Relative Error	$MRE = \displaystyle{{\mathop \sum \nolimits_i \left| {{y_i} - \widehat {{y_i}}} \right|} \over {\mathop \sum \nolimits_i {y_i}}}$
${RME}$	Relative Mean Errors	$RME = \displaystyle{1 \over N}\mathop \sum \limits_{i = 1}^N \left| {\displaystyle{{{y_i} - \widehat {{y_i}}} \over {{y_i}}}} \right|$
MAD	Mean Absolute Deviation	$MAD = \displaystyle{1 \over N}\mathop \sum \limits_{i = 1}^N \left| {{y_i} - \widehat {{y_i}}} \right|$
RE	Relative Error	$RE = \displaystyle{{{y_i} - \widehat {{y_i}}} \over {{y_i}}}\; \times 10$
Where N indicates the total number of observation/samples. ${\hat {\bi {y}}_{i}}$ is the predicted value and ${\bi{y}_\bi{i}}$ is the actual travel /ground truth. The error between the predicted and actual values is presented by ${\bi{\varepsilon }_{i}}$ = $\hat {\bi{y}_{i}} - {\bi{y}_{i}}$. The mean of method output and observations are expressed as:$\bar {\bi y} = \displaystyle{1 \over {N}}\mathop {\sum \nolimits_{\bi{i} = 1}^{\bi N}} \hat {\bi{y}_{i}}$ $\bar {\bi y} = \displaystyle{1 \over \bi{N}}\mathop \sum \nolimits_{\bi{i} = 1}^{\bi N} {\bi{y}_{i}}$.		

Van Lint (2004) presented an evaluation framework where the reliability of an approach is evaluated in terms of its accuracy, validity, robustness, and adaptiveness. The consideration of all these factors indicates that an acceptable ATP/TTP method should obtain accurate results under different traffic situations as well as in the presence of missing or erroneous data. However, most of the researchers have focused on evaluating the accuracy of the methods by using different statistical errors. These error measures obtain the average error of a method throughout the study and are therefore not sufficient to evaluate the robustness of the method under different situations (Van Hinsbergen, Van Lint & Sanders, 2007). The assessment of models can be enhanced if the error measuring statistics are computed separately for different periods or even for different traffic situations.

**Figure 11:**
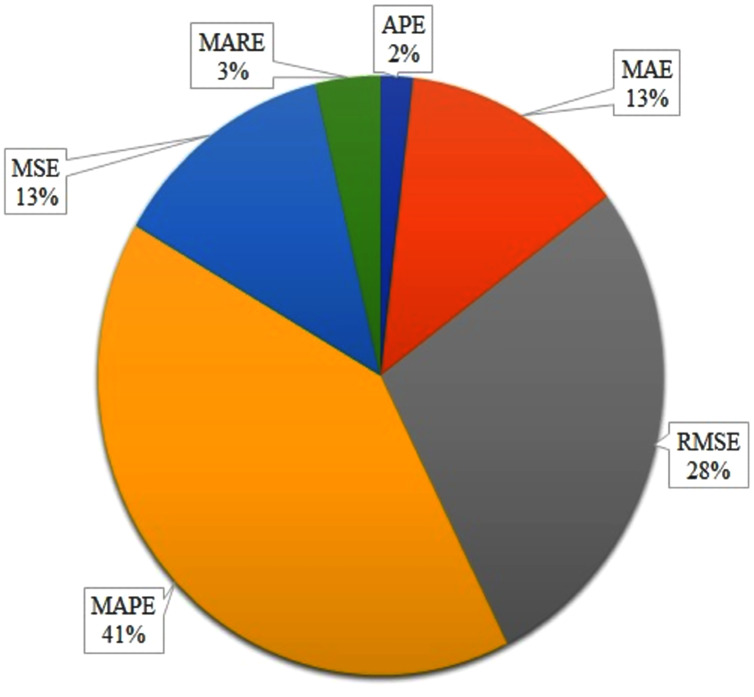
Most commonly used evaluation metrics for ATP and TTP.

**Figure 12:**
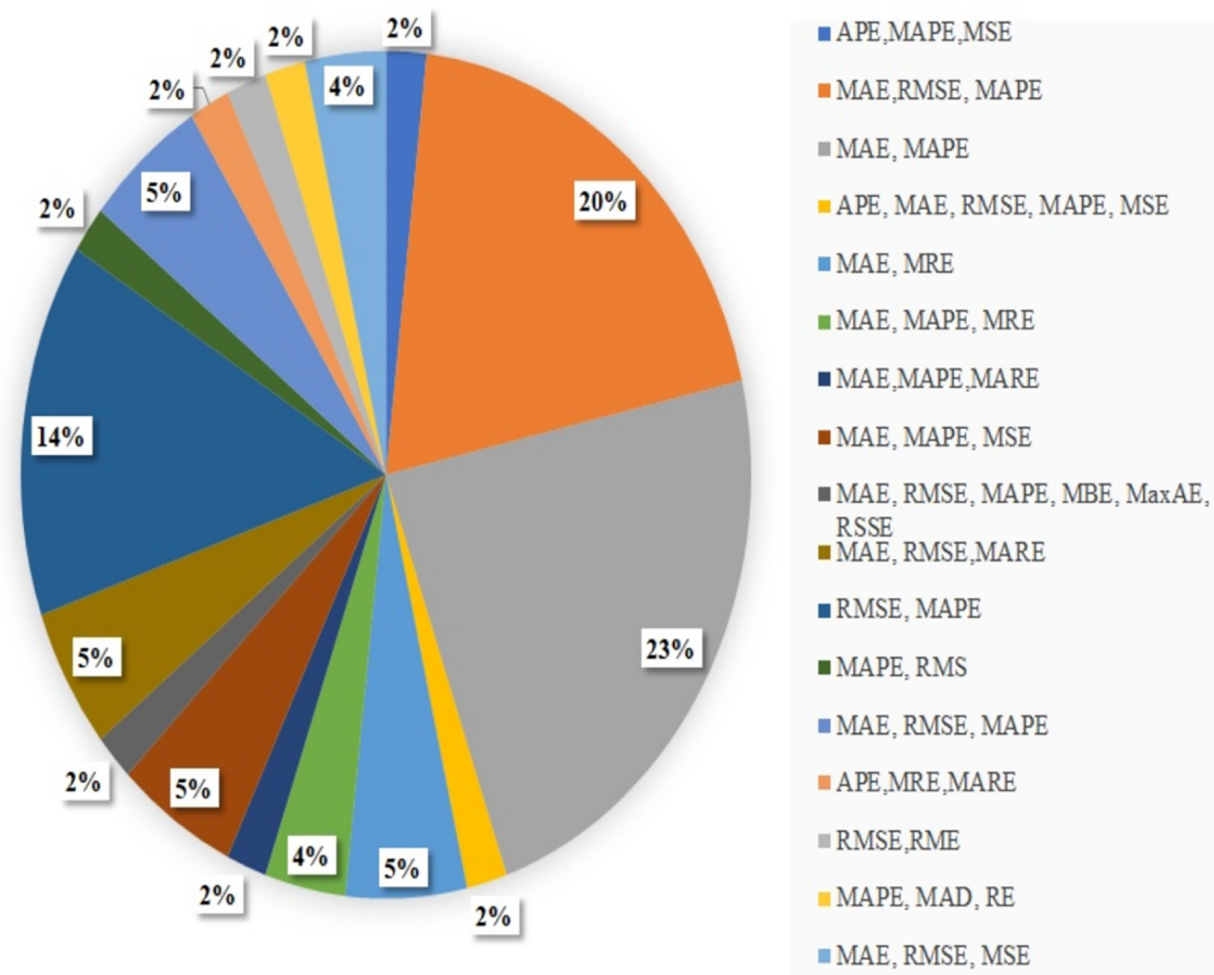
Distribution of combined evaluation metrics in TTP and ATP.

Our literature review shows that APE, MAE, RMSE, MAPE, MSE as well as MARE are the six major evaluation metrics used in the arrival/travel time predictions. Figures 11 and 12 present the extent to which each of the metrics is used at both the individual level or at the combined levels (a combination of different metrics). The frequency usage of these metrics among the papers reviewed in this study is presented in the following figures with 41%, 28%, 13%, 2%, 3% and 13% MAPE, RMSE, MAE, APE, MARE, and MSE respectively (Figure 11). The result is extended by presenting the frequencies of all the combined evaluation metrics that involve APE, MAPE and MSE; MAE, RMSE and MAPE; MAE and MAPE; APE, MAE, RMSE, MAPE and MSE, etc. for the ATP/TTP. According to the result, as shown in Figure 12, it can be observed that combining MAE, RMSE, MAPE (20%), MAE, MAPE (23%) and RMSE, MAPE (14%) are the most used evaluation metrics.

Therefore, the combination of MAE, RMSE and MAPE as evaluation metrics for ATP or TTP approach helps in achieving optimal performance of the methods in the papers we have reviewed.

## Limitations

Some limitations should be considered regarding this study: (1) the search query, in our study, the large number of papers have been assessed and reviewed. It indicates that our review is revealing an adequate understanding of the state of the art in the field. Nevertheless, it is worth noting that the findings of this study are confined to the selected papers extracted from various database and review papers. Therefore, the reference lists of books, chapters and theses can be also considered for systematic review. (2) our study only classified different factors into five groups: ‘*Spatial factors*’, ‘*Temporal factors*’, ‘*Traffic information*’, ‘*Driving style*’, and ‘*Augmented features*’. However, it did not determine which group would be more effective to the ATP and TTP.

## Implications

Recent technological advancements have allowed for the development of various intelligent transportation systems, which can be used to enhance the performance of transportation systems. Consequently, the knowledge in the field of ITS, specifically, the knowledge on different aspects of ATP and TTP models could be beneficial. For this reason, this paper aims to provide an overview of recently developed systems for ATP and TTP. Furthermore, it presents evaluation metrics, different factors and dataset. We hope that this study enables ITS designers and developers to have a holistic understanding of different techniques. As a result of our findings, certain recommendations can be given to future developers and researchers in the field of the intelligent transportation system. Moreover, this review can serve as a source of systematic information for the research community in the ITS domain and would be helpful for the research community.

## Conclusion and Future Work

Transport systems play a key role in advanced societies nowadays. In this paper, we conducted a literature review study of travel and arrival time prediction models. The research scope we investigated is restricted to road networks. Our review has focused on several aspects which can influence traffic management and control. Intelligent traffic management and control could be one of the solutions to tackle the challenge of increasing travel demand, CO2 emissions, safety concerns, and wasted fuels. Travel and arrival time are very useful indicators of traffic and are vital components of intelligent transportation systems. Therefore, we notice considerable research interest in modelling and predicting these traffic indicators.

After a four-phase search process, we selected and analyzed 115 papers. A complete list of the selected papers is presented in Tables 1 and 2. The review findings could benefit researchers in the field to set out and align their research agendas. Moreover, further research directions arising from this study concern the better models for ATP and TTP. However, in this study, we investigate travel and arrival time based on the various techniques in detail. We present a complete classification of the existing approaches for the prediction of travel and arrival time. This review reveals that a prediction approach can be categorized into historical data, statistical-based methods, machine learning-based methods or Hybrid methods. We have also investigated the factors that influence arrival and travel time. Furthermore, we have discussed several dataset resources to use in modelling. Moreover, we indicate and list the advantages and disadvantages of the proposed methods along with their evaluation metrics.

We observe from the examined publications that 56% of the total papers on arrival time prediction relied on a machine learning method, 2% on a historical-based technique and 42% on a statistical-based technique. On the other hand, the distributions of the methods used for TTP techniques are 32% for statistical methods, 4% for historical-based methods and 64% for machine learning-based methods. Furthermore, we also present the rate at which the factors are employed based on the main groups of the factors: Temporal Factors (47%), Traffic Information (26%), Spatial Factors (20%), Augmented Factors (6%) and Personalized Information (%1). The result of our review shows that APE, MAE, RMSE, MAPE, MSE as well as MARE are the 6 major evaluation metrics used in the arrival time and travel time predictions.

**As future work,** we would like to present some solutions to overcome shortcomings of existing methods, and highlight significant research challenges in the following directions:

***Artificial intelligence-based method***—it has been noted that an AI-based method is a robust model, can manage a large amount of data, learns the potential patterns from data and is a fast computation tool. However, few methods have been proposed for TTP and ATP using an AI method. Hence, there is a need to develop prediction systems using AI model such as ANN, CNN, LSTM etc.

***Hybrid methods***—the combination of different type of methods to enhance and tackle the performance and limitation of each participant method. A fusion may enable a combination method to tackle any unexpected situation, which might be useful for ATP and TTP. For instance, Li & Chen (2014) propose a combination of K-means clustering, decision trees and neural networks to predict travel time on a freeway with non-recurrent congestion. However, to create a fusion model knowledge of the characteristics of the existing methods is necessary, which leads to the following research direction.

***Comparative study of ATP and TTP methods***—this study must provide a complete explanation of the approaches and their qualities to choose a method for each situation. Furthermore, in future research, the effects of different types of factors on ATP and TTP performance could be tested.

***Social media contributions***—the traffic situation reports from social media (*e.g*., Twitter) can be added as an augmented dataset to predict travel/arrival time more accurately.

***Unexpected change of traffic condition***—a major limitation of the existing method is that they are not accurate when dealing with changes due to unexpected traffic. Therefore, future work could focus on rapidly detecting non-recurrent congestion that can adapt to unexpected traffic situations.

***Integration of dataset resource***—most of the papers we studied used only one type of dataset resource as input to the model and very few models incorporated different types of datasets. A single unique dataset may not adequately represent the traffic situation. Therefore, a combination of different kinds of datasets can be used to enhance the accuracy of the arrival/travel prediction method.

***The fusion of traffic and augmented information***—several factors affect the performance and quality of ATP and TTP methods in addition to the traffic information (*e.g*., incident, signals, dwelling time, etc.). Factors including the behaviours of the drivers, meteorology, information about roads or maps, etc. which must be included and considered in the models. Furthermore, travel/arrival time varies over time and space; hence, a method that considers both spatial and temporal variations together may capture the traffic situations better, and thereby can be an effective way to reduce the uncertainty of ATP/TTP results. Moreover, most of the proposed methods have not considered the impact of driving behaviour. Travel/arrival time is not only affected by traffic conditions but also affected by driving behaviour (**e.g*., driving experience, driver preferences*). As a result, two drivers may have different travel/arrival times for the same segment/link/road significantly. Hence, it is more applicable and practical to analyse and predict personalized travel/arrival time for the different drivers. However, previous research has only employed a few traffic parameters to develop their models. Therefore, as future work, other rich information/traffic parameters can be considered (need to be collected and analysed) to improve predictive operations.

## Additional Information and Declarations

### Data Availability

The following information was supplied regarding data availability: This Literature Review did not use any data.
